# Identification and characterisation of tertiary lymphoid organs in human type 1 diabetes

**DOI:** 10.1007/s00125-021-05453-z

**Published:** 2021-04-29

**Authors:** Éva Korpos, Nadir Kadri, Sophie Loismann, Clais R. Findeisen, Frank Arfuso, George W. Burke, Sarah J. Richardson, Noel G. Morgan, Marika Bogdani, Alberto Pugliese, Lydia Sorokin

**Affiliations:** 1 Institute of Physiological Chemistry and Pathobiochemistry, University of Muenster, Muenster, Germany; 2 Cells-in-Motion Interfaculty Centre, University of Muenster, Muenster, Germany; 3 Science for Life Laboratory, Department of Medicine, Karolinska Institute, Karolinska University Hospital, Solna, Stockholm Sweden; 4 Present Address: School of Human Sciences, The University of Western Australia, Perth, WA Australia; 5 Department of Surgery, Division of Transplantation, Leonard M. Miller School of Medicine, University of Miami, Miami, FL USA; 6 Institute of Biomedical & Clinical Science, University of Exeter, Exeter, UK; 7 Matrix Biology Program, Benaroya Research Institute, Seattle, WA USA; 8 Diabetes Research Institute, Leonard M. Miller School of Medicine, University of Miami, Miami, FL USA; 9 Division of Endocrinology and Metabolism, Department of Medicine, Leonard M. Miller School of Medicine, University of Miami, Miami, FL USA; 10 Department of Microbiology and Immunology, Leonard M. Miller School of Medicine, University of Miami, Miami, FL USA

**Keywords:** Autoimmunity, Basement membrane, Fibroblastic reticular cells, High endothelial venules, Lymph node, Reticular fibres, Tertiary lymphoid organs, Transplantation, Type 1 diabetes

## Abstract

**Aims/hypothesis:**

We and others previously reported the presence of tertiary lymphoid organs (TLOs) in the pancreas of NOD mice, where they play a role in the development of type 1 diabetes. Our aims here are to investigate whether TLOs are present in the pancreas of individuals with type 1 diabetes and to characterise their distinctive features, in comparison with TLOs present in NOD mouse pancreases, in order to interpret their functional significance.

**Methods:**

Using immunofluorescence confocal microscopy, we examined the extracellular matrix (ECM) and cellular constituents of pancreatic TLOs from individuals with ongoing islet autoimmunity in three distinct clinical settings of type 1 diabetes: at risk of diabetes; at/after diagnosis; and in the transplanted pancreas with recurrent diabetes. Comparisons were made with TLOs from 14-week-old NOD mice, which contain islets exhibiting mild to heavy leucocyte infiltration. We determined the frequency of the TLOs in human type 1diabetes with insulitis and investigated the presence of TLOs in relation to age of onset, disease duration and disease severity.

**Results:**

TLOs were identified in preclinical and clinical settings of human type 1 diabetes. The main characteristics of these TLOs, including the cellular and ECM composition of reticular fibres (RFs), the presence of high endothelial venules and immune cell subtypes detected, were similar to those observed for TLOs from NOD mouse pancreases. Among 21 donors with clinical type 1 diabetes who exhibited insulitis, 12 had TLOs and had developed disease at younger age compared with those lacking TLOs. Compartmentalised TLOs with distinct T cell and B cell zones were detected in donors with short disease duration. Overall, TLOs were mainly associated with insulin-containing islets and their frequency decreased with increasing severity of beta cell loss. Parallel studies in NOD mice further revealed some differences in so far as regulatory T cells were essentially absent from human pancreatic TLOs and CCL21 was not associated with RFs.

**Conclusions/interpretation:**

We demonstrate a novel feature of pancreas pathology in type 1 diabetes. TLOs represent a potential site of autoreactive effector T cell generation in islet autoimmunity and our data from mouse and human tissues suggest that they disappear once the destructive process has run its course. Thus, TLOs may be important for type 1 diabetes progression.

**Graphical abstract:**

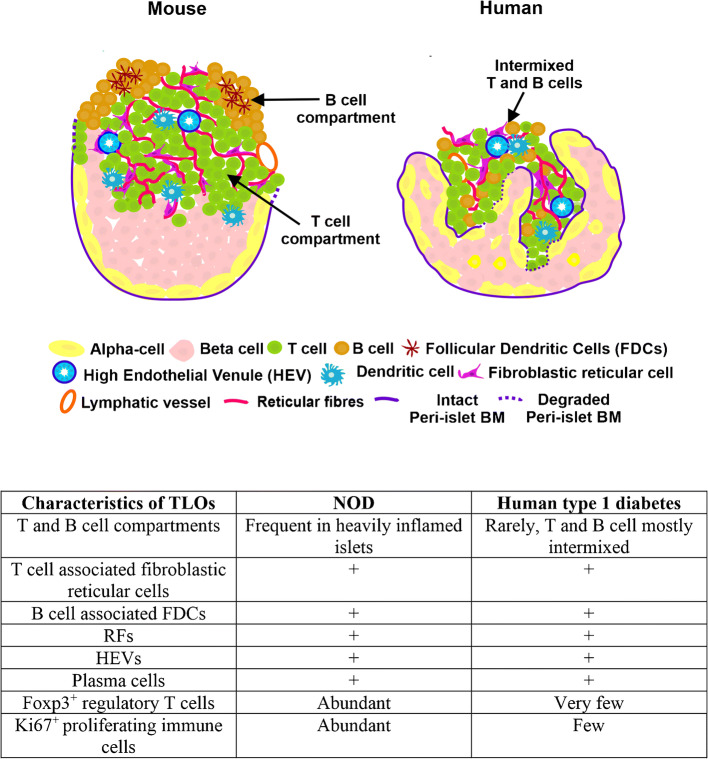

**Supplementary Information:**

The online version contains peer-reviewed but unedited supplementary material available at 10.1007/s00125-021-05453-z.

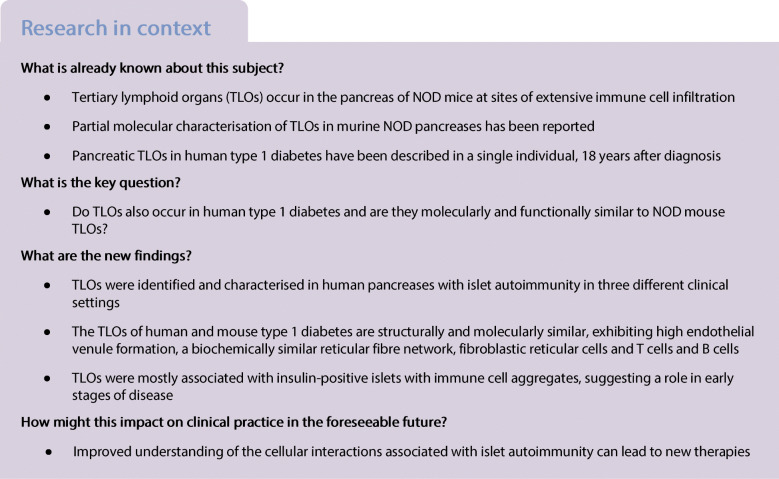



## Introduction

Tertiary lymphoid organs (TLOs), highly organised structures compartmentalised into T cell and B cell zones by a reticular fibre (RF) network, form in inflamed tissues during chronic infection, autoimmunity and cancer [1]. The impact of TLOs on disease varies. During acute infection TLOs support the immune response and clearance of pathogens [2, 3] and in cancer TLOs support or suppress the immune response against tumour cells, depending on tumour type [4]. TLOs worsen the severity of most autoimmune diseases [5, 6].

TLOs show structural and functional similarities to secondary lymphoid organs, even though they form after birth. TLOs generate local immune responses in chronically inflamed tissues and are not surrounded by a fibrous capsule [7]. Like lymph nodes (LNs), TLOs are characterised by an extensive RF network, consisting of a unique inner core of fibrillar collagens surrounded by basement membrane (BM) proteins and enclosed in a sheath of fibroblastic reticular cells (FRCs) [8, 9]. TLOs contain high endothelial venules (HEVs), specialised postcapillary venules [10] through which CCR7^+^ T cells and naive B cells (CCR7^low^) are recruited from the circulation to the site of inflammation [11]. Like LNs, mature TLOs display clear compartmentalisation of T cells and B cells, with higher RF density in T cell zones compared with B cell zones. T cells are supported by FRCs and the B cells by follicular dendritic cells (FDCs) [12]. These features of RFs, FRCs and FDCs allow distinction between immature and mature TLOs even in the absence of staining for B cells and T cells. RFs act as conduits for the transport of small molecules and soluble antigens (<70 kDa) from peripheral sites of inflammation to HEVs, required for the rapid recruitment of lymphocytes [8, 13, 14]. Hence, RFs promote inflammation.

TLOs were described in the pancreas of NOD mice, a model of autoimmune type 1 diabetes [15–17]. There are no published studies reporting direct evidence of TLOs in the pancreas of humans with type 1 diabetes, except for a recent case report of a person aged 66 years who had developed type 1 diabetes 18 years earlier, at age 48 [18]. We investigated the existence of TLOs in human pancreases with islet autoimmunity in three different clinical settings: preclinical, defined by the expression of at least two disease-associated autoantibodies (aAbs); clinically diagnosed type 1 diabetes [19]; and recurrent diabetes in transplanted pancreas [20, 21]. Using a unique repertoire of cellular and extracellular matrix (ECM) markers, we compared TLOs in human and NOD mouse pancreas.

## Methods

### Human samples

We studied pancreas sections from organ donors without diabetes and from donors with ongoing islet autoimmunity in three distinct clinical settings of type 1 diabetes: preclinical; clinically diagnosed; and recurrent disease in the transplanted pancreas. Key clinical and laboratory characteristics of the individuals examined are listed in Table 1.

**Table 1:** Clinical characteristics of the organ donors

Donor characteristic	Donor no.	Age (years)	Sex	Age at type 1 diabetes diagnosis (years)	Diabetes duration (years)	Antibody status				Insulitis	C-peptide (nmol/l)
						GADA	IA-2A	ZnT8A	mIAA		
Non-diabetic aAb^−^ (*n* = 5)	nPOD 6098	17.8	M	n/a	n/a	–	–	–	–	–	0.46
	nPOD 6230	16	M	n/a	n/a	–	–	–	–	–	1.72
	nPOD 6339	23.3	M	n/a	n/a	–	–	–	–	–	3.49
	nPOD 6335	18.8	M	n/a	n/a	–	–	–	–	–	2.93
	nPOD 6430	27.1	M	n/a	n/a	–	–	–	–	–	3.68
Non-diabetic single aAb^+^ (*n* = 7)	nPOD 6027	18.8	M	n/a	n/a	–	–	+	–	–	n/a
	nPOD6123	23.2	F	n/a	n/a	+	–	–	–	–	0.66
	nPOD6151	30	M	n/a	n/a	+	–	–	–	–	1.81
	nPOD6181	31.9	M	n/a	n/a	+	–	–	–	–	0.19
	nPOD6301	26	M	n/a	n/a	+	–	–	–	–	1.29
	nPOD 6310	28	F	n/a	n/a	+	–	–	–	+	3.48
	nPOD 6314	21	M	n/a	n/a	+	–	–	–	–	0.49
Non-diabetic multiple aAb^+^ (*n* = 6)	nPOD 6080	69.2	F	n/a	n/a	+	–	–	+	–	0.60
	nPOD 6158	40.3	M	n/a	n/a	+	–	–	+	–	1.85
	nPOD 6167	37	M	n/a	n/a	–	+	+	–	–	1.79
	nPOD 6197	22	M	n/a	n/a	+	+	–	–	+	5.78
	nPOD 6267	23	F	n/a	n/a	+	+	–	–	+	5.49
	nPOD 6450	22	F	n/a	n/a	+	–	+	–	+	1.80
Donors with type 1 diabetes (*n* = 24)	nPOD 6362	24.9	M	24.9	0	+	–	–	–	+	0.12
	nPOD 6228	18	M	18	0	+	+	+	–	+	0.03
	EADB E405	8	F	7.98	<0.019	n/a	n/a	n/a	n/a	+	n/a
	EADB SC115	1.25	F	1.24	0.008	n/a	n/a	n/a	n/a	+	n/a
	EADB E124B	17	M	16.98	0.02	n/a	n/a	n/a	n/a	+	n/a
	EADB E308	3	F	2.92	0.08	n/a	n/a	n/a	n/a	+	n/a
	nPOD6209	5	F	4.75	0.25	–	+	+	+	+	0.03
	nPOD 6414	23.1	M	22.67	0.43	+	–	+	+^a^	+	0.05
	nPOD 6247	24	M	23.4	0.6	–	–	–	+^a^	+	0.15
	nPOD 6052	12	M	11	1	+	–	–	–	+	0.05
	nPOD 6224	21	F	19.5	1.5	–	–	–	–	+	<0.01
	nPOD 6342	14	F	12	2	+	–	–	+^a^	+	0.08
	nPOD 6371	12.5	F	10.5	2	+	+	+	+^a^	+	0.03
	nPOD 6396	17.1	F	15.1	2	–	–	–	–	+	0.01
	nPOD 6195	19.3	M	14.3	5	+	+	+	+^a^	+	0.01
	nPOD 6212	20	M	15	5	+	–	–	–	+	0.01
	nPOD 6243	13	M	8	5	–	–	–	+^a^	+	0.13
	nPOD 6306	19	M	14	5	–	–	–	+^a^	+	0.01
	nPOD 6325	20	F	14	6	+	+	–	+^a^	+	0.04
	nPOD 6070	22.6	F	15.6	7	+	–	–	+^a^	+	0.01
	nPOD 6245	22	M	15	7	+	+	–	–	+	0.01
	nPOD 6302	38.5	M	6	32.5	–	–	–	–	–	0.05
	nPOD 6085	71	F	8	63	–	–	–	+*^a^	–	0.01
	nPOD 6086	89	F	5	84	–	–	–	–	–	0.01

^a^If the individuals have been on exogenous insulin for more than 10 days the mIAA is not a reliable marker for autoimmunity

F, female sex; M, male sex; mIAA, micro IAA

Specifically, the Network for Pancreatic Organ Donors with Diabetes (nPOD; www.JDRFnPOD.org) provided pancreas cryosections from the following groups:

- five organ donors without diabetes and negative for type 1 diabetes-associated autoantibodies (aAb^−^ control group);
- thirteen organ donors positive for one (*n* = 7) or more (*n* = 6) type 1 diabetes-associated autoantibodies, of which 1/7 and 3/6, respectively, had insulitis;
- twenty organ donors with type 1 diabetes (additionally, paraffin sections from four autopsies were provided by the Exeter Archival Diabetes Biobank [EADB; https://foulis.vub.ac.be/]). Among these 24 donors, 21 had insulitis and nine had disease duration shorter than 6 months. The disease duration among those with insulitis was 0–7 years. This group included three donors with long disease duration (32.5–83 years) who lacked insulitis and insulin staining in the islets;
- three individuals with long-standing type 1 diabetes who experienced recurrence of disease in the transplanted pancreas several years after successful simultaneous pancreas and kidney (SPK) transplantation, despite chronic immunosuppression (Table 2). All pancreas transplant biopsies were from individuals who displayed insulitis and residual islets with insulin-positive beta cells and these individuals had the cardinal features of recurrent type 1 diabetes we previously described [20].

**Table 2:** Clinical characteristics of donors where recurrent type 1 diabetes developed after SPK transplantation

Characteristic	Donor no.		
	nPOD 3626	nPOD 3678	nPOD 3681
Sex	M	M	M
Age at type 1 diabetes onset, years	29	14	12
Age at transplant, years	43	39	35
Ab status at transplant			
GADA	−	+	−
IA-2A	+	−	−
ZnT8A	−	−	−
Age at biopsy, years	63	49	40
Ab status at biopsy			
GADA	+	+	−
IA-2A	+	+	+
ZnT8A	−	+	+
HbA_1c_ at biopsy, mmol/mol (%)	115.3 (12.7)	94.5 (10.6)	93.4 (10.7)
Age at biopsy, years	63	49	40
Time from transplant to diabetes recurrence, years	17.2	8	4.9
Duration of diabetes recurrence at biopsy, years	1.4	1.4	0.6

Ab, antibody; M, male sex

All tissue donors were de-identified and samples were obtained with the necessary ethical approvals.

### Immunofluorescence microscopy

Immunofluorescence staining of pancreas cryosections was performed as described [22]. Sections were fixed in methanol at −20°C, washed, blocked with 1% BSA in PBS and incubated overnight at 4°C with primary antibody diluted in blocking solution. After washing, sections were incubated overnight at 4°C with secondary antibodies. Paraffin sections were rehydrated and submitted to antigen retrieval by heating the sections for 30 min in 10 mmol/l citrate buffer, pH 6.0 in a microwave (700 W). Sections were treated with Pronase (Roche, Germany) (1 mg/ml Pronase in 50 mmol/l Tris-HCl [pH 7.5] and 5 mmol/l EDTA) for 15 min at 37°C to retrieve masked ECM molecules. Blocking and incubation with the primary and secondary antibodies were performed as for cryosections. The primary antibodies employed are listed in electronic supplementary material (ESM) Table 1. Human thymus and LN sections were used to validate the primary antibodies. The specificity of secondary antibodies was verified by omitting the primary antibodies from the staining procedure (ESM Fig. 1). The sections were examined using a Zeiss AxioImager (Zeiss, Germany) or an LSM700 microscope (Zeiss).

### Quantification of TLOs in type 1 diabetes human samples

Pancreas cryosections stained for pan-laminin (PLM), CD45 and insulin were used to count insulin-positive and insulin-negative islets (ESM Fig. 2) and associated aggregated or intermixed CD45^+^ cell infiltrates. Paraffin sections were stained for collagen type VI instead of PLM since the antigen retrieval for PLM was not compatible with cell surface staining. Serial sections were stained for MECA79 to visualise HEVs [10]. CD3 and CD20 staining was used to identify T cells and B cells. The density of RFs identified by PLM and the presence of FDCs helped to differentiate immature TLOs from mature TLOs in the absence of specific staining for T cells and B cells in donor no. 6362. Insulitis was defined according to the consensus definition given by Campbell-Thompson et al [23] as at least 15 CD45^+^ cells adjacent or within three islets per section. Control, non-diabetic donor samples were not included in quantification since insulitis was not detected in any of them. To assess TLOs in relation to disease severity, we classified pancreatic islets into four categories representing different phases of the disease process: phase 0 (normal, insulin positive, no insulitis); phase 1 (insulin positive, with insulitis); phase 2 (insulin negative with insulitis); and phase 3 (insulin negative, no insulitis, also known as pseudo-atrophic islets).

### Electron microscopy

Samples for electron microscopy were prepared according to standard protocols [24] and analysed with an electron microscope (EM-410; Philips, the Netherlands).

### Animals

NOD mice (Bomholtgaard, Ry, Denmark) were screened for diabetes by urine analyses of glucosuria (Combur3 Test; Roche). The mice were housed in the animal facility of the Institute of Physiological Chemistry and Pathobiochemistry, University of Muenster, on a 12 h light–dark cycle, and were fed with regular diet and given water ad libitum. Animal experiments followed Swedish and German animal welfare guidelines. Fourteen-week-old female NOD mice (*n* = 6) were used, since at this age all severity stages of inflamed islets are found in the pancreas. Mice were killed by cervical dislocation. Organs were frozen and cut by cryotome.

### Statistical analysis

The significance of the difference between two or more groups of data was evaluated using the Mann–Whitney *U* test and the Kruskal–Wallis test, respectively. Correlation analysis was performed using the non-parametric Spearman’s rank correlation test. Contingency analysis was performed using χ^2^ (and Fisher’s exact) test. *p* < 0.05 was considered statistically significant. All analyses were performed using GraphPad Prism version 9.00 for Windows (GraphPad Software, San Diego, CA, USA).

As far as possible, the NIH guidelines for reporting of experimental conditions were employed. However, randomisation and blinded assessment of samples were not possible because of the limited numbers of human samples of defined conditions available and the need for their fast use upon arrival from nPOD.

## Results

We examined pancreas specimens from donors with islet autoimmunity and/or type 1 diabetes (Tables 1 and 2) provided by the nPOD [25] and EADB repositories [26]. We used immunofluorescence staining and confocal microscopy to assess markers of TLO formation, including RFs, HEVs, chemokines and immune cell aggregates (ESM Table 1). Comparisons were made to pancreatic TLOs from NOD mice, and studies included in-depth characterisation of RFs in NOD mice.

### TLOs in the human pancreas with islet autoimmunity in donors with type 1 diabetes

**Fig. 1:**
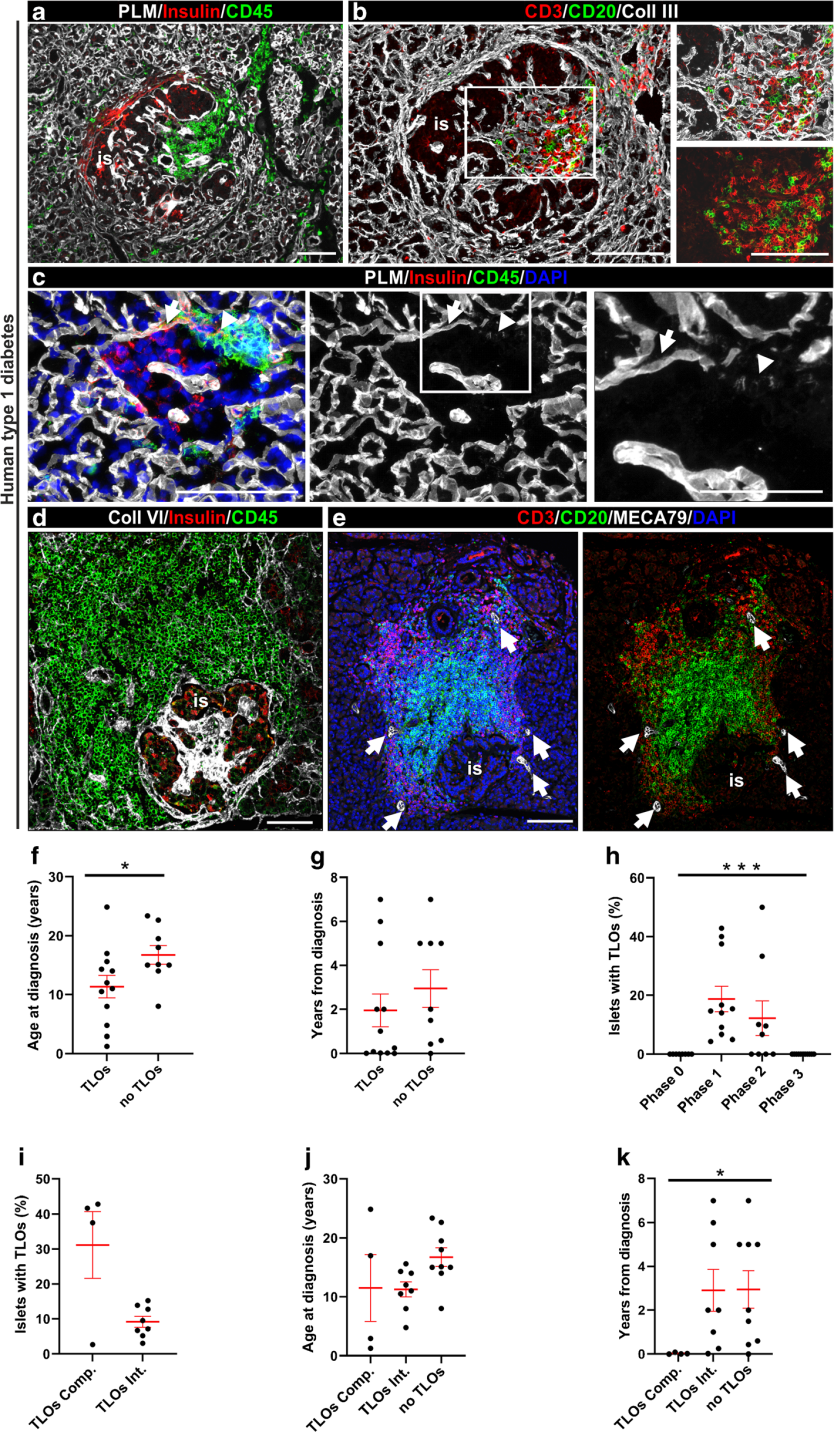
TLOs in human donors with type 1 diabetes. (**a**, **b**) Representative images of a heavily inflamed (CD45^+^) insulin-positive islet in peri-insulitis stage (donor no. 6325) (**a**) with intermixed T cells and B cells (**b**). PLM marks the peri-islet BM, endothelial BM of blood vessels and acinar BM (**a**) and a consecutive section stained for T cells (CD3^+^), B cells (CD20^+^) and collagen type III shows the RFs (**b**); boxed area is shown at higher magnification. (**c**) Representative image of a heavily inflamed islet characterised by disruption of peri-islet BM (PLM^+^) by infiltrating immune cells (CD45^+^). The arrow marks intact peri-islet BM and the arrowhead marks disrupted peri-islet BM; boxed area is shown at higher magnification. (**d**, **e**) Visualisation of a heavily inflamed insulin-positive islet with T cell and B cell compartments (donor no. E124B). Collagen VI marks the interstitial matrix of the islet and surrounding exocrine tissue, CD45 labels all leucocytes (**d**). Immunofluorescence staining for CD3 and CD20 reveals T cell and B cell compartments and MECA79 staining identifies HEVs (arrows) in the T cell zone (**e**). (**f**, **g**) Frequency of TLOs in relation to the age at diabetes diagnosis (**f**, *p<0.05) and disease duration (**g**, *p*=0.3). (**h**) Proportion of islets with TLOs in relation to disease severity, which was defined by assigning the islets into four phases based on their insulin content and on the presence of insulitis: phase 0 (insulin-positive islets without insulitis); phase 1 (insulin-positive islets with insulitis); phase 2 (insulin-negative islets with insulitis); and phase 3 (insulin-negative islets without insulitis). There was a statistically significant difference across these groups using the Kruskal–Wallis test (***p<0.001). Proportion of islets with TLOs in donors with compartmentalised (TLOs Comp.) and intermixed TLOs (TLOs Int.) (*p*=0.2) (**i**). There was no difference in age at diagnosis among donors classified by TLO stages (*p*=0.1090, Kruskal–Wallis test) (**j**) but disease duration was different (*p<0.05, Kruskal–Wallis test) (**k**). Scale bars, 100 μm, or 50 μm for areas of higher magnification. Data are shown as means ± SD in all graphs. Coll, collagen; is, islet

Of the 24 donors with clinically diagnosed type 1 diabetes, 21 had insulitis and a diabetes duration of 0–7 years (Table 1). Twelve of these 21 donors had pancreatic TLOs as revealed by staining for T cells and B cells, RFs and MECA79 (Fig. 1a, b, d, e). T cells and B cells appeared to be intermixed in immature TLOs (Fig. 1b) and organised into T cell and B cell compartments in mature TLOs (Fig. 1e). The peri-islet BM appeared to be intact in peri-insulitis lesions where immune cells accumulated at one pole of the islet (Fig. 1b) and was breached at sites where immune cells penetrated the islet (Fig. 1c). TLOs were associated with insulin-positive islets in 7/12 donors, with both insulin-positive and insulin-negative islets in 4/12 donors; in a single donor, TLOs were rarely associated with insulin-negative islets (Table 3). The mean age of diagnosis was significantly lower among donors with TLOs compared with those without TLOs (mean ± SD: 11.35 ± 6.59 vs 16.74 ± 4.76 years, *p* < 0.05, Fig. 1f); however, there was no significant difference in disease duration (Fig. 1g). The frequency of TLOs was significantly different according to disease severity, with the highest frequency found in islets with insulitis (phase 1 and 2 islets) (Fig. 1h, *p* < 0.001, Kruskal–Wallis test). When analysing the islets with insulitis among the 12 donors with TLOs and type 1 diabetes, there was an inverse correlation between the frequency of islets with TLOs and age of onset and disease duration (ESM Fig. 3), although this did not reach statistical significance; in this analysis, we calculated frequencies for phase 1 and 2 islets, phases when TLOs were observed. Of note, 39/383 (10.18%) insulin-positive islets with insulitis had TLOs compared with 10/204 (4.9%) insulin-negative islets with insulitis (*p* = 0.0276, χ^2^ [and Fisher’s exact] test).

**Table 3:** Distribution of pancreatic TLOs within type 1 diabetes donors

Donor no.	Age of onset (years)	Disease duration (years)	No. of islets examined	Phase 0 islets (insulin^+^ islets, no insulitis)			Phase 1 islets (insulin^+^ islets with insulitis)			Phase 2 islets (insulin^−^ islets with insulitis)			Phase 3 islets (insulin^−^ islets, no insulitis)		
				Without TLOs	With TLOs	% with TLOs	Without TLOs	With TLOs	% with TLOs	Without TLOs	With TLOs	% with TLOs	Without TLOs	With TLOs	% with TLOs
Donors with insulitis and TLOS with compartmentalised T cells and B cells															
nPOD 6362	24.90	0	255	0	0	n/a	110	5	4.35	75	0	0.00	65	0	0.00
EADB SC115	1.24	0.01	111	1	0	0.00	6	4	40	1	1	50	98	0	0.00
EADB E124B	16.98	0.02	56	33	0	0.00	5	3	37.50	0	0	n/a	15	0	0.00
EADB E308	2.92	0.08	125	65	0	0.00	4	3	42.86	0	0	n/a	53	0	0.00
Mean	11.51	0.03	136.75	24.75	0.00	0.00	31.25	3.75	31.18	19.00	0.25	12.50	57.75	0.00	0.00
SD	11.37	0.03	84.27	30.90	0.00	0.00	52.51	0.96	18.02	37.34	0.50	25	34.27	0.00	0.00
Donors with insulitis and TLOs with intermixed T cells and B cells															
EADB E405	7.98	0.02	71	32	0	0.00	14	1	6.67	0	0	n/a	24	0	0.00
nPOD6209	4.75	0.25	23	4	0	0.00	5	1	16.67	8	0	0.00	5	0	0.00
nPOD 6052	11	1	77	0	0	n/a	22	4	15.38	9	1	10.00	41	0	0.00
nPOD 6342	12	2	53	0	0	n/a	10	1	9.09	22	0	0.00	20	0	0.00
nPOD 6371	10.50	2	48	2	0	0.00	37	6	13.95	2	1	33.33	0	0	n/a
nPOD 6195	14.30	5	256	0	0	n/a	0	0	n/a	57	6	9.52	193	0	0.00
nPOD 6325	14	6	221	99	0	0.00	96	5	4.95	14	1	6.67	6	0	0.00
nPOD 6070	15.6	7	84	33	0	0.00	35	6	14.63	6	0	0.00	4	0	0.00
Mean	11.27	2.91	104.13	21.25	0.00	0.00	27.38	3.00	10.17	14.75	1.13	7.44	36.63	0.00	0.00
SD	3.58	2.71	85.61	34.48	0.00	0.00	30.75	2.51	5.95	18.41	2.03	11.36	64.64	0.00	0.00
Donors with insulitis and without TLO															
nPOD 6228	18	0	36	0	0	n/a	24	0	0.00	10	0	0.00	2	0	0.00
nPOD 6414	22.67	0.43	87	2	0	0.00	27	0	0.00	35	0	0.00	23	0	0.00
nPOD 6247	23.4	0.6	87	0	0	n/a	4	0	0.00	38	0	0.00	45	0	0.00
nPOD 6224	19.5	1.5	81	3	0	0.00	5	0	0.00	25	0	0.00	48	0	0.00
nPOD 6396	15.1	2	77	3	0	0.00	27	0	0.00	25	0	0.00	22	0	0.00
nPOD 6212	15	5	136	9	0	0.00	5	0	0.00	22	0	0.00	100	0	0.00
nPOD 6243	8	5	64	0	0	n/a	5	0	0.00	28	0	0.00	31	0	0.00
nPOD 6306	14	5	28	6	0	0.00	8	0	0.00	3	0	0.00	11	0	0.00
nPOD 6245	15	7	134	0	0	n/a	0	0	n/a	20	0	0.00	99	0	0.00
Mean	16.74	2.94	81.06	2.56	0.00	0.00	11.67	0.00	0.00	22.89	0.00	0.00	42.33	0.00	0.00
SD	4.76	2.56	37.07	3.17	0.00	0.00	10.98	0.00	0.00	11.07	0.00	0.00	35.53	0.00	0.00

Most of the TLOs showed mixed T cell and B cell aggregates (8/12 donors) (Fig. 1b). TLOs with compartmentalised T cell and B cell areas were detected in only four donors with type 1 diabetes (Fig. 1d, e, Table 3 and ESM Fig. 4); however, in these samples TLOs with intermixed B cells and T cells predominated and compartmentalised TLOs represented 20–33% of the total TLOs. The frequency of TLOs was not statistically different in donors with compartmentalised TLOs compared with those with intermixed TLOs (Fig. 1i). We observed no significant differences in the mean age at diabetes diagnosis of donors with compartmentalised vs intermixed TLOs or those lacking TLOs (Fig. 1j). Disease duration was significantly shorter in donors with compartmentalised TLOs vs donors with intermixed TLOs and vs donors with no TLOs, respectively (mean ± SD duration: 0.03 ± 0.035, 2.909 ± 2.709 years and 2.948 ± 2.564, respectively; *p* < 0.05, Fig. 1k). When we compared donors with vs without TLOs we found no significant differences in the positivity rates for each aAb (GAD aAb [GADA], tyrosine phosphatase-related islet antigen 2 aAb [IA-2A], zinc transporter 8 aAb [ZnT8A]; excluding insulin aAb [IAA], not tested) or for multiple aAbs (ESM Fig. 5) by Fisher’s exact test.

### Potential TLOs in the human pancreas with islet autoimmunity from aAb^+^ donors without diabetes

**Fig. 2:**
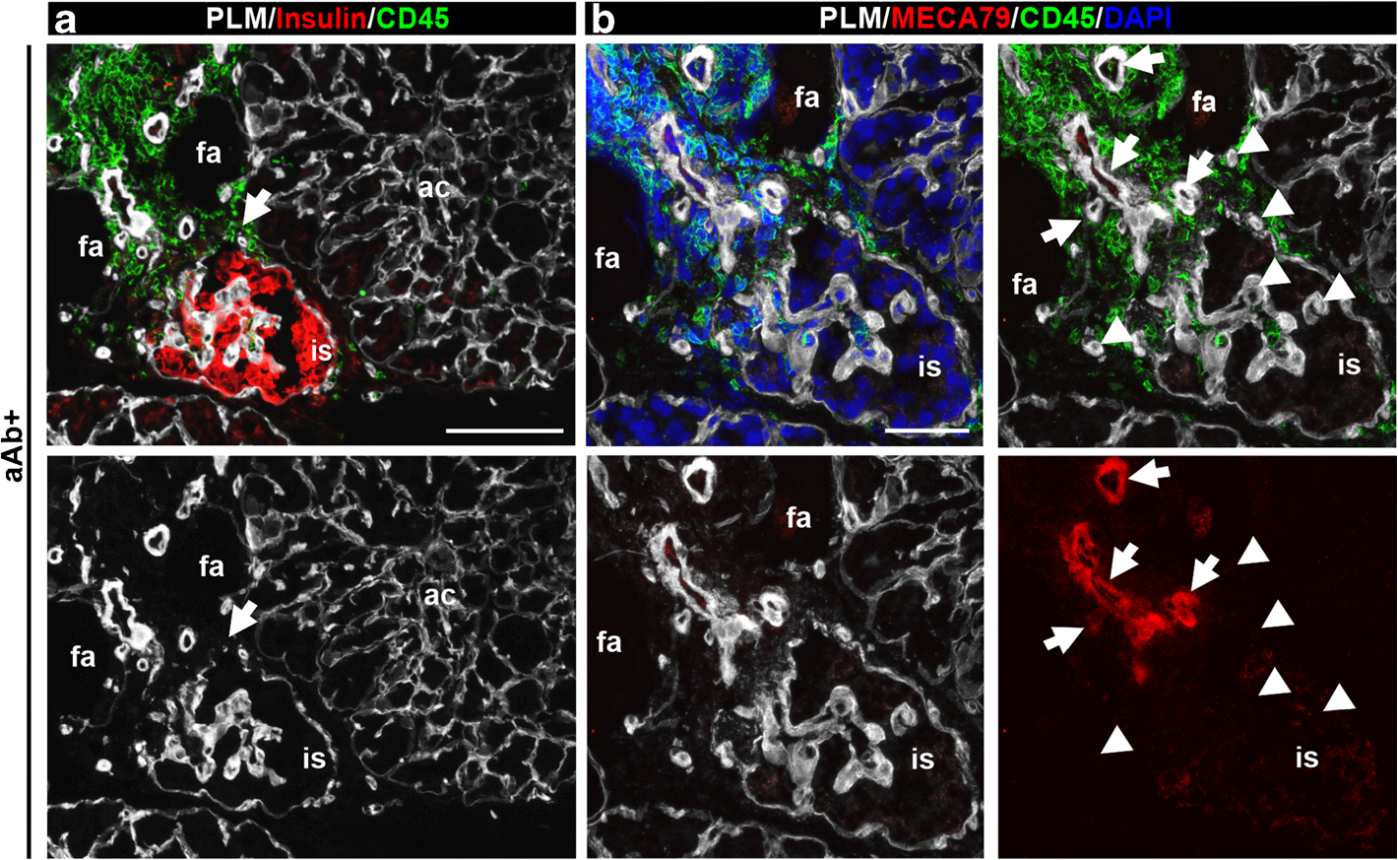
Potential TLOs in preclinical human donors with type 1 diabetes. Representative images of a double-aAb^+^ donor (no. 6197) with insulin-positive inflamed islet stained for PLM, insulin and CD45 (**a**) or MECA79, PLM and CD45 (**b**). Arrows mark the disruption of peri-islet BM in (**a**) and the MECA79^+^ HEVs in (**b**). Arrowheads point to MECA79^−^ vessels. Scale bars, 100 μm (**a**) and 50 μm (**b**). ac, acini; fa, fat; is, islet

Among the organ donors positive for one or two aAbs, insulitis was observed in 1/7 and 3/6 samples, respectively, and was associated with insulin-positive islets in all donors examined. In the three double-aAb^+^ donors with insulitis, we performed triple staining for PLM, CD45 and insulin (Fig. 2a) or for CD45, PLM and MECA79 (Fig. 2b). The latter revealed peri-islet CD45^+^ immune cell aggregates surrounding MECA79^+^ HEVs, suggesting the existence of TLOs. Given the limited number of pancreas sections available, we could not perform CD3/CD20 staining for aAb^+^ donors. However, the staining combinations allowed us to demonstrate insulitis and the presence of TLOs, which by their features we consider to be immature. Such leucocyte aggregates and MECA79^+^ HEVs were not detected in the single-aAb^+^ donor with insulitis, in which we analysed 29 islets with insulitis. No beta cell loss was apparent in any of the aAb^+^ donors.

### TLOs in pancreas of recipients who experienced recurrent type 1 diabetes following the transplant

**Fig. 3:**
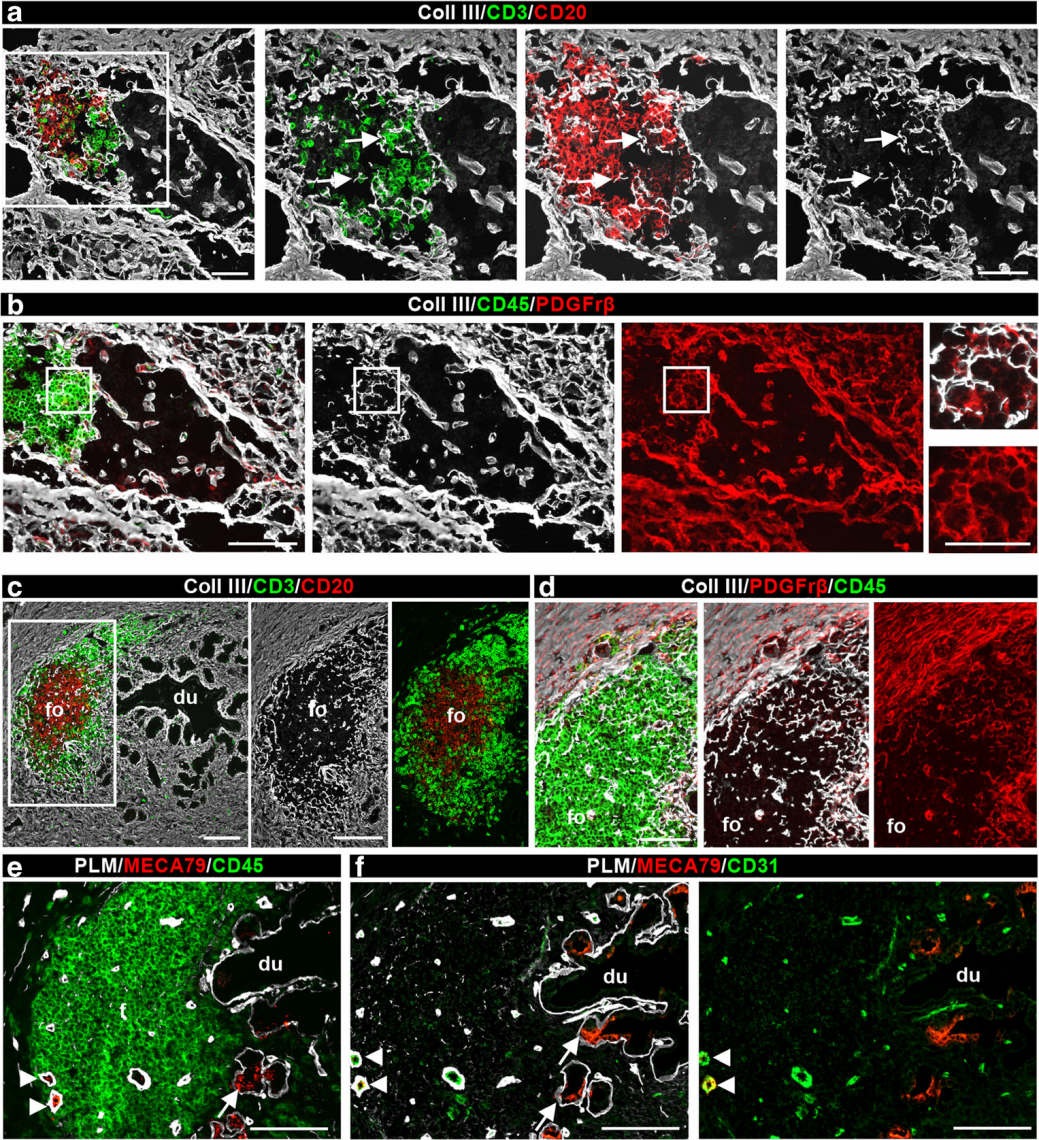
TLO detection in pancreas transplanted into recipients in whom recurrent type 1 diabetes developed. (**a**, **b**) Representative immunofluorescence images of pancreatic TLOs associated with islets in a biopsy sample (donor no. 3678). Triple immunofluorescence staining for CD3^+^ T cells, CD20^+^ B cells and collagen III for RFs (arrows) and the interstitial matrix of the islet and exocrine pancreas are shown (**a**). A parallel section was stained for PDGFrβ^+^ FRCs associated with the collagen III^+^ RFs (**b**); CD45 staining marks leucocytes. Boxed areas are shown at higher magnification. (**c**, **d**) Triple immunofluorescence staining using the same antibody combination as in (**a**) and (**b**) reveals TLO in the wall of the pancreatic duct. Boxed area is shown at higher magnification. (**e**, **f**) Triple staining for PLM, MECA79 and CD45 or CD31 revealing MECA79 staining of HEVs (arrowheads) and of some epithelial cells in the pancreatic ducts (arrows). Scale bars, 50 μm (**a**–**c**, **e**, **f**) or 25 μm (inset in **b**, **d**). Coll, collagen; du, duct; fo, follicle

We previously reported that about 5–6% of individuals with type 1 diabetes who receive SPK transplantation develop recurrence of disease in the transplanted pancreas; this typically occurs several years after transplantation despite chronic immunosuppression and in the absence of clinical rejection [20, 21]. Here, we examined biopsies containing insulin-positive islets and exhibiting insulitis from three transplant recipients in whom evidence of acute pancreas rejection was lacking (Table 2) [20, 21]. TLOs were detected in all three biopsies. Immunofluorescence staining for collagen III and CD45 or CD20 and CD3 revealed leucocyte infiltration around insulin-positive islets (Fig. 3a, b) that was associated with platelet-derived growth factor receptor β (PDGFrβ) staining (Fig. 3b). All three biopsies showed some degree of T cell and B cell organisation (Fig. 3a); in one sample (from donor no. 3678) we detected B cell follicle-like structures surrounded by T cells in close association with pancreatic ducts (Fig. 3c). We detected PDGFrβ^+^ FRCs surrounding the RFs (Fig. 3d) and MECA79^+^ HEVs (Fig. 3e, f) in the T cell areas.

### TLOs associated with pseudo-atrophic islets and pancreatic ducts

**Fig. 4:**
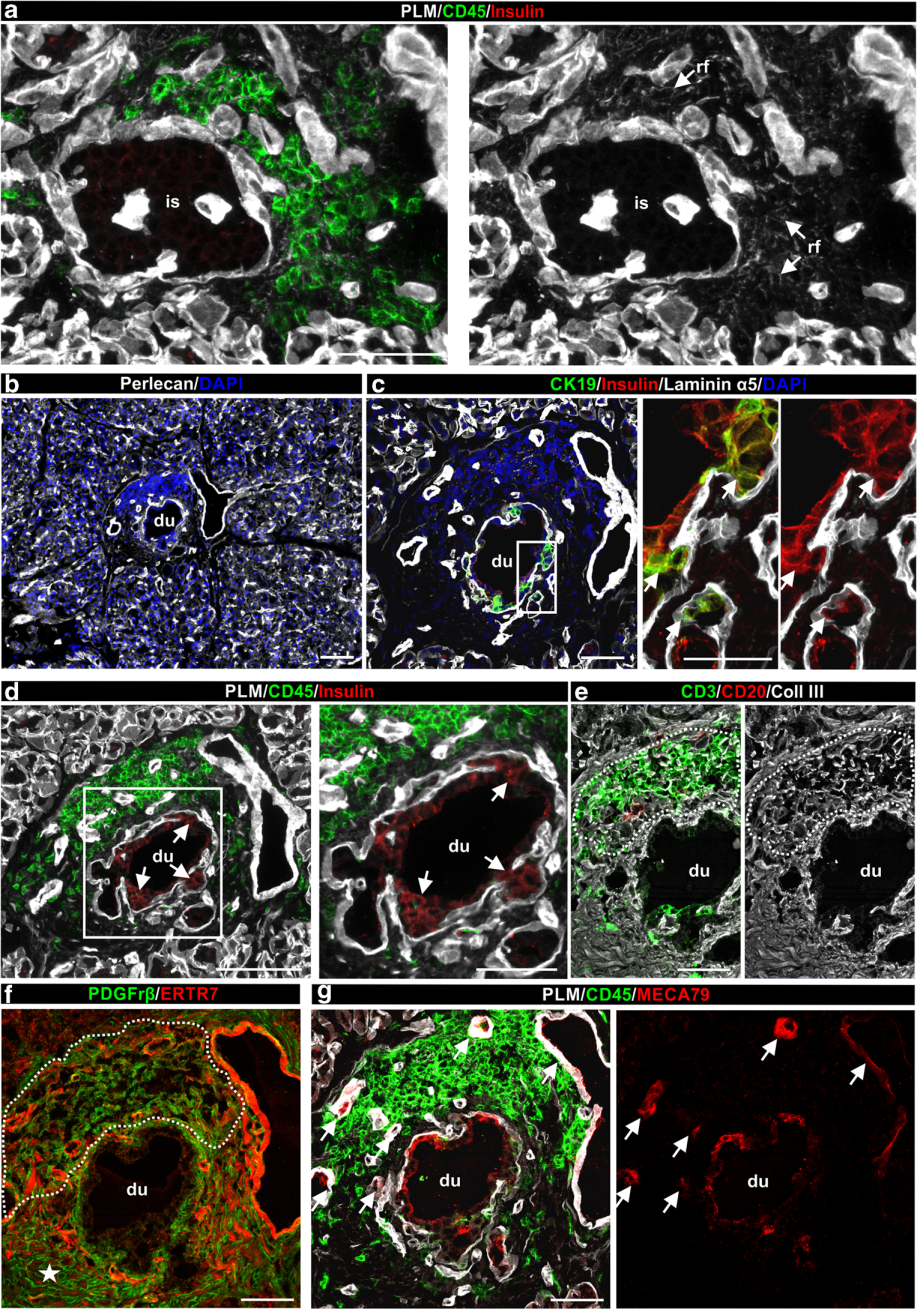
TLOs associated with insulin-negative islets and with the wall of pancreatic ducts in human type 1 diabetes. (**a**, **d**) Representative immunofluorescence images of pancreas sections (from donor no. 6195) triple-stained for insulin, PLM and CD45 showing inflamed pancreatic islet (**a**) and pancreatic duct (**d**). Arrows point to RFs (**a**) and to insulin-positive cells in the pancreatic duct (**d**). (**b**) Low magnification image of a consecutive section of the duct stained for BM marker perlecan and DAPI to visualise the surroundings of the inflamed duct. There was no islet detected in close vicinity to the duct. (**c**) Triple immunofluorescence for CK19, insulin and laminin α5 to mark ductal epithelial cells, beta cells and BMs, respectively. Boxed area is shown at higher magnification. Arrows in (**c**) mark insulin-positive cells among the pancreatic duct epithelial cells. (**e**) Immunofluorescence staining of pancreatic duct for CD3^+^ T cells, CD20^+^ B cells and collagen type III to mark RFs. (**f**) Double staining of pancreatic duct for PDGFrβ^+^ and ERTR7. Note the different PDGFrβ staining pattern at sites of leucocyte accumulation (dashed line) compared with the non-inflamed region of the duct (star). (**g**) Staining for MECA79 reveals HEVs (arrows) in inflamed pancreatic duct and some signal on the surface of the duct epithelium. Scale bars, 100 μm (**a**, **b**, **d**); 50 μm (**c**, **e**–**g**; and **d** [area shown at higher magnification]) or 25 μm (**c** [area shown at higher magnification]). Coll, collagen; du, duct; is, islet; rf, RFs

In five donors with type 1 diabetes (no. 6052, no. 6195, no. 6325, no. SC115 and no. 6371), TLOs were detected in association with insulin-negative pseudo-atrophic islets (Fig. 4a, ESM Fig. 4d) and in close association with pancreatic ducts containing insulin-positive cells (Fig. 4b–d). The insulin-positive cells in the duct showed some co-staining for CK19, a marker of epithelial ductal cells (Fig. 4c). No islets were detected in close vicinity to the ducts and there was no indication of pancreatitis based on histological characterisation by nPOD. Duct-associated TLOs had mostly T cells and little or no CD20^+^ B cells (Fig. 4e). Dense collagen III^+^/ERTR7^+^ RFs associated with PDGFrβ^+^ cells were observed in the T cell infiltrates (Fig. 4f). Furthermore, MECA79^+^ HEVs were identified at sites of leucocyte accumulation in the wall of the pancreatic duct (Fig. 4g).

### Characteristics of TLOs in the human pancreas with islet autoimmunity compared with NOD mice

**Fig. 5:**
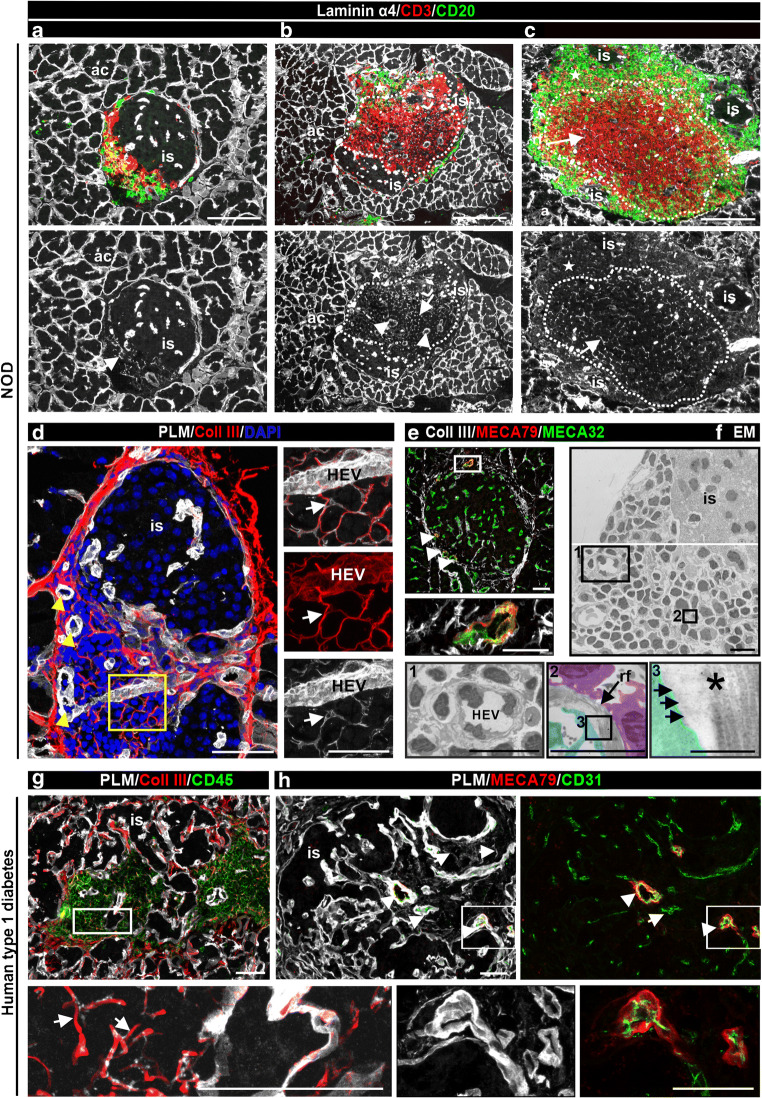
Comparison of pancreatic TLOs in NOD mouse and human type 1 diabetes samples. (**a**–**c**) Triple immunofluorescence staining of 14-week-old NOD mouse pancreases for CD3 to label T cells, CD20 to mark B cells and laminin α4 to mark the islet BMs, acinar BMs and the BM of RFs revealed B cells and T cells intermixed (**a**) and compartmentalised TLOs (**b**, **c**). Dashed lines mark T cell areas, stars mark B cell zones; arrowheads label HEVs and arrows mark RFs. ‘is’ indicates the healthy part of the islet. (**d**, **g**) Immunofluorescence staining for collagen III to label the core of RFs and PLM to mark BMs of inflamed islets of NOD mouse (**d**) and human type 1 diabetes samples (**g**). Arrowheads point to HEVs and arrows indicate the RFs (**d**). Boxed areas are shown at higher magnification. (**e**, **h**) MECA79 staining reveals HEVs in pancreas from NOD mouse (**e**, arrowhead) and human type 1 diabetes samples (**h**). All blood vessels are marked either with MECA32 (**e**) or CD31 (**h**) and PLM stains the BM of RFs and pancreatic islets. (**f**) Electron micrograph of an inflamed islet shows the ultrastructure of HEV (box 1) and of RFs (box 2). FRCs (pseudo-coloured in cyan) and lymphocytes (pseudo-coloured in violet) closely associate with RFs (box 2), which is shown at higher magnification in box 3; asterisk labels the inner collagen fibres and arrows mark the outer BM. The same micrograph with grid is shown in ESM Fig. 7. Boxed area is shown at higher magnification. Scale bars, 100 μm (**a**–**c**), 50 μm (**d**, **e**, **g**, **h**), 25 μm (**d**, **e** [areas shown at higher magnification]), 5 μm (**f** [boxes 1 and 2]) or 500 nm (**f** [box 3]). ac, acini; Coll, collagen; EM, electron micrograph; is, healthy islet; rf, RFs

Comparisons between mouse and human TLOs revealed similarities and differences. All stages of TLOs and insulitis could be detected in 14-week-old NOD mice [22], ranging from intermixed T cells and B cells (Fig. 5a) to well-organised T cell and B cell compartments (Fig. 5b, c) with a dense RF network in the T cell zone (Fig. 5c), as also occurs in mouse LNs (ESM Fig. 6). In contrast, infiltrating T cells and B cells were intermixed in most of the human pancreas samples with islet autoimmunity and TLOs with T cell and B cell compartments were rarely detected (detected in 4/12 donors) (Table 3, Fig. 1d, e and ESM Fig. 4).

**Fig. 6:**
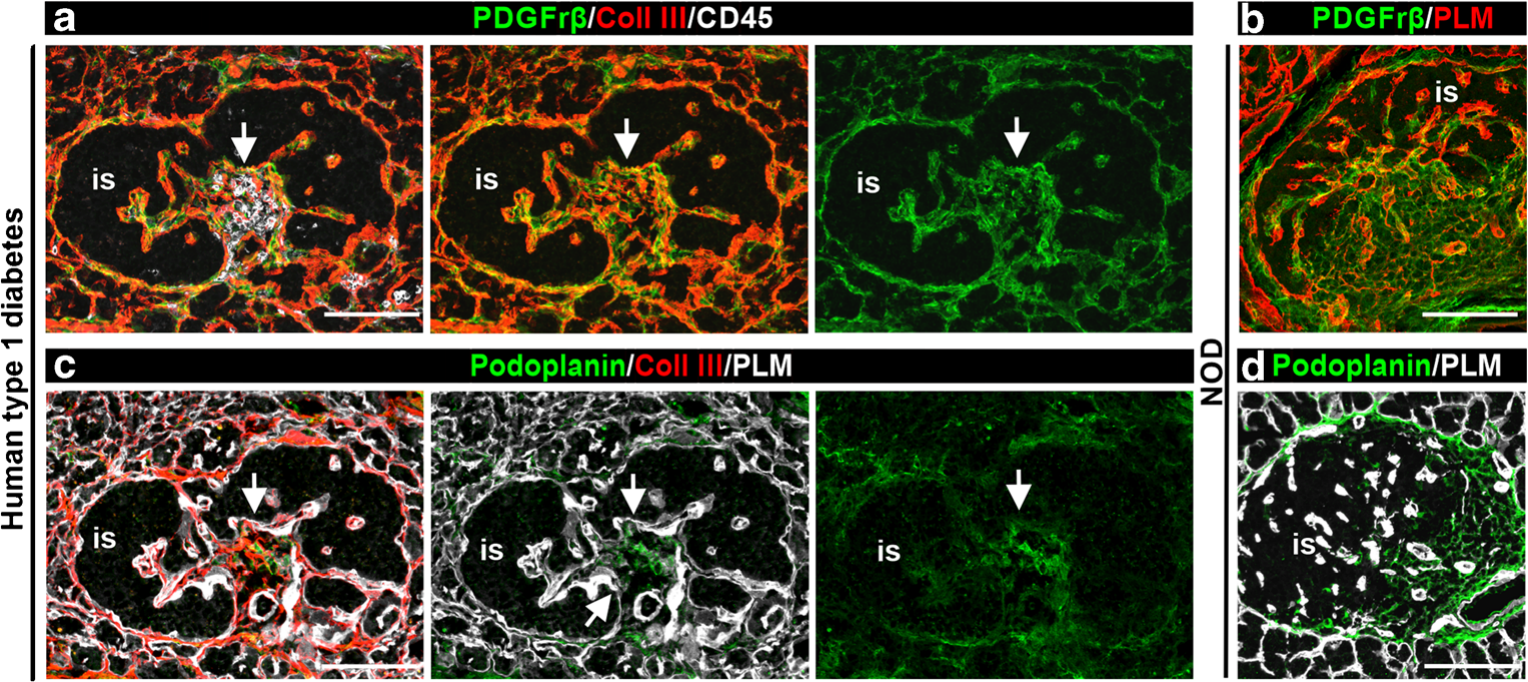
Comparison of fibroblastic reticular cell markers in pancreatic TLOs in NOD mouse and human type 1 diabetes samples. (**a**, **c**) Immunofluorescence staining of inflamed human pancreas (donor no. 6325) for collagen III, PDGFrβ and CD45 (**a**) or collagen III, podoplanin and PLM (**c**). (**b**, **d**) Immunofluorescence staining of NOD mouse pancreases for PLM and PDGFrβ (**b**) or podoplanin (**d**). Arrows mark accumulation of FRCs at site of inflammation. Scale bars, 100 μm. Coll, collagen; is, healthy part of the islet

### Characteristics of RFs in the human pancreas with islet autoimmunity compared with NOD mice

Studies in LNs have revealed a well-organised RF network composed of RFs and FRCs [8, 13, 27], which provides physical and functional support for immune cells (ESM Fig. 6) [28]. The same features were described in pancreatic TLOs of NOD mice [17]. RFs of NOD mice and human pancreatic TLOs stained for BM molecules and fibrillar collagen type III (Fig. 5a–g). TLOs contained a filigree RF network, as shown by laminin α4 and PLM staining in mouse (Fig. 5a–d) and human samples (Fig. 5g, h), HEVs defined by MECA79 staining (Fig. 5e, h), and a thick BM [8] (Fig. 5b, h). Electron microscopy of an inflamed NOD mouse islet confirmed the presence of fibrillar collagen bundles in the RF core, covered by a thin BM and surrounded by lymphocytes and FRCs (Fig. 5f and ESM Fig. 7), consistent with studies of LNs [8]. We conducted in-depth characterisation of the structural components of RFs in NOD mice (ESM Results and ESM Fig. 8) using a large repertoire of antibodies specific for ECM molecules (ESM Table 1). RFs in inflamed human islets have the same basic structure as RFs of pancreatic TLOs in NOD mice.

### Conduit function of RFs

RFs of LNs can act as conduits for the rapid transport of soluble, low-molecular-weight molecules such as chemokines and antigens [8, 13]. We investigated whether the RFs of pancreatic TLOs have a similar function. Tracer experiments using FITC-labelled dextran and immunofluorescence staining for chemokines and insulin (as antigen) support a potential conduit function of RFs in pancreatic TLOs, similar to their function in LNs (ESM Results and ESM Fig. 9).

### FRCs in pancreatic TLOs

Several FRC markers have been described, including PDGFrβ and podoplanin; the latter is also a lymphatic marker [29]. Triple staining of human samples revealed a strong PDGFrβ signal within CD45^+^ infiltrates where it occurred surrounding the collagen III RFs (Fig. 6a), consistent with the location of FRCs in mouse pancreatic TLOs (Fig. 6b) and LNs [17, 29]. Podoplanin staining was limited to lymphatic vessels (not shown) in NOD mouse and human pancreases but was present in inflamed islets in pancreases of both humans with type 1 diabetes (Fig. 6c) and NOD mice (Fig. 6d). Our data suggest that stromal cells associated with RFs in human pancreatic TLOs are similar to FRCs described previously in LNs and TLOs of NOD mice.

### Immune cell subtypes and proliferating cells in pancreatic TLOs

**Fig. 7:**
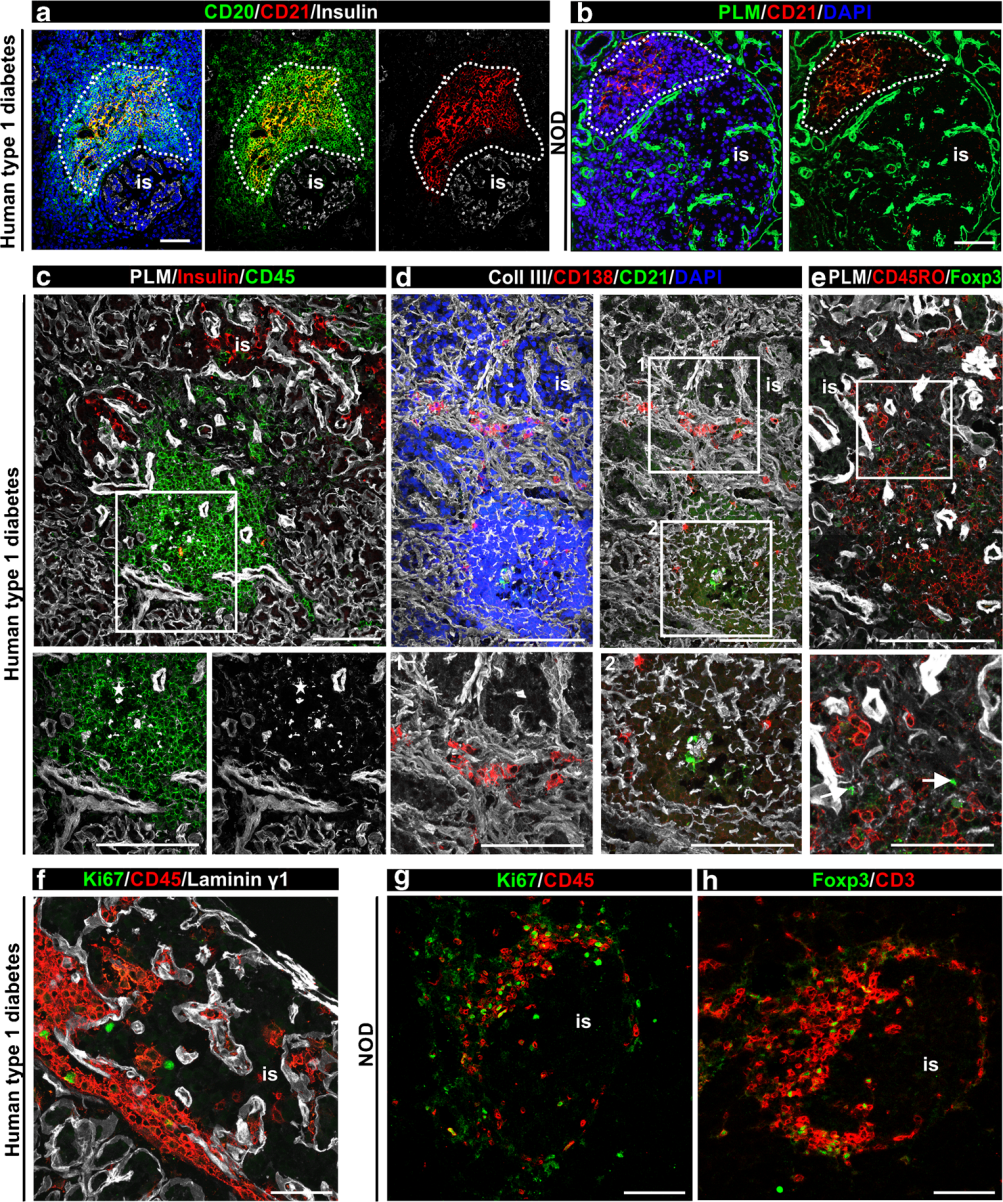
Comparison of FDCs and immune cell subtypes in pancreatic TLOs in NOD mouse and human type 1 diabetes samples. (**a**, **b**) Triple immunofluorescence staining for CD21, CD20 and insulin (**a**), or PLM, CD21 and DAPI (**b**) reveals CD21^+^ FDCs in B cell follicles in inflamed human (donor no. E124) (**a**) and NOD mouse islets (**b**), dashed line marks the B cell compartment. (**c**–**e**) Triple immunofluorescence staining of consecutive sections of an extensively inflamed insulin-positive islet (donor no. 6362) for PLM, CD45 and insulin (**c**), collagen III, CD138 to mark plasma cells and CD21 to label FDCs (**d**) and PLM and FOXP3 to mark regulatory T cells and CD45RO to mark memory T cells (**e**). Star indicates the B cell follicle (**c**) and arrow points to FOXP3+ regulatory T cell (**e**, inset). Boxed areas are shown at higher magnification. (**f**, **g**) Immunofluorescence staining for the proliferation marker Ki67, CD45 and laminin γ1 to mark all BMs of human (donor no. 6362) (**f**) and NOD mouse (**g**) inflamed islets reveals few proliferating immune cells. (**h**) FOXP3 staining of NOD mouse samples reveals regulatory T cells. Scale bars, 50 μm, (25 μm for areas shown at higher magnification). Coll, collagen; is, islet

In the four type 1 diabetes samples exhibiting compartmentalisation of T cells and B cells in association with insulin-positive (from donors no. 6362, no. E308 and no. E124B) and insulin-negative islets (from donor no. SC115) (Table 3 and ESM Fig. 4), the T cell compartment contained a dense RF network visualised by collagen VI staining and the B cell zone showed less-dense RFs and the presence of CD21^+^ FDCs (Fig. 7a and ESM Fig. 4), similarly to inflamed islets from NOD mice (Fig. 7b). FDCs are non-migratory cells associated only with B cell follicles in LNs [30]; their detection suggests the formation of germinal centres and propagation of the immune response [31]. Therefore, we investigated immune cell subtypes that are indicative of an ongoing inflammatory reaction: CD138^+^ plasma cells were detected in human TLOs scattered throughout CD45^+^ areas (Fig. 7c), in close proximity to islets (Fig. 7d); memory T cells (CD45RO^+^) were abundant (Fig. 7e); forkhead box P3 (FOXP3)^+^ regulatory T cells were rarely detected (none, or one or two FOXP3^+^ cells/TLO, Fig. 7e). Few Ki67^+^/CD45^+^ proliferating cells were found in inflamed human islets (Fig. 7f). Plasma cells and memory T cells were also detected in NOD mouse samples [32, 33], similarly to Ki67^+^ cells (Fig. 7g) and FOXP3^+^ (Fig. 7h), which were abundant in inflamed mouse islets, consistent with earlier reports [32, 33]. Quantification within the inflamed islet from donor no. 6362 (Fig. 7) shows 6% plasma cells, 0.5% FOXP3^+^ T cells, 53% memory T cells and 2% Ki67^+^ cells among the CD45^+^ cells. These results suggest that TLOs may contribute to the long-term perpetuation of inflammation in human type 1 diabetes.

## Discussion

Previous studies associated pancreatic TLOs with type 1 diabetes in NOD mice [15, 16]. An earlier investigation failed to identify these structures using immunofluorescence staining for CD4^+^ T cells and CD19^+^ B cells in pancreases donated by four humans with type 1 diabetes (12–22 years old, 1–8 years of diabetes duration) [15]. Recently, TLOs were described in the pancreas of a single person with long disease duration [18]. Our study is the first to systematically examine pancreatic TLOs in a cohort of donors (*n* = 37) at distinct stages of islet autoimmunity. We provide definitive evidence for the existence of TLOs in the human pancreas of individuals at high risk of diabetes, at/after diagnosis, and in pancreases of recipients who experienced recurrent type 1 diabetes after transplantation. The clear identification of TLOs in our study also arises from the assessment of multiple ECM components of the RF network, different leucocyte types, stromal cells and specialised endothelial markers. However, TLO positive islets are a rare event and as such the comparisons between different groups described in this study should be interpreted with caution. Collectively, our data suggest that the occurrence of TLOs correlates with leucocyte infiltrates surrounding mostly insulin-positive islets. We also show several structural similarities between LNs and the pancreatic TLOs, and between TLOs in human and NOD mouse pancreases. The findings support the concept that TLOs in the pancreas of individuals with islet autoimmunity/type 1 diabetes may support recruitment and activation of lymphocytes from the circulation and thereby promote disease progression.

A strength of our study is that we could examine pancreas tissue from donors with recent-onset type 1 diabetes, residual insulin-positive islets and ongoing autoimmunity. We examined 24 donors with clinical type 1 diabetes, most of whom were selected for having insulitis (21/24) and several of whom had very short disease duration. Among the 21 type 1 diabetes samples exhibiting insulitis, 12 contained TLOs; the donors of samples with TLOs were diagnosed at a significantly younger age than those without TLOs, while disease duration was not statistically different. We also show that TLOs form in the transplanted pancreas in recipients with recurrent type 1 diabetes, suggesting that they may contribute to reappearance of the disease in these individuals, who also had circulating autoreactive T cells and autoantibodies despite chronic immunosuppression to prevent rejection [20, 34].

The detection of insulitis and TLOs in a significant proportion of multiple autoantibody-positive donors suggests that TLO formation may precede clinical diagnosis, as in NOD mice, and supports a role for TLOs in promoting inflammation at early stages of diabetes. Given the extreme rarity of donors with a single aAb and insulitis, we cannot determine whether TLOs are present at this stage.

Insulitis among the donors with islet autoimmunity was not as extensive as the insulitis observed in NOD mice; this finding was expected, based on earlier comparisons of mouse and human pancreas pathology [35, 36]. TLOs in human samples mostly resembled those seen in NOD mice exhibiting mild insulitis, where the immune cells accumulated around the islets (peri-insulitis) and T cells and B cells were intermixed. Consequently, the typical features of TLOs, such as HEVs, RF network and the intermixed T cells and B cells, were mostly localised to peri-islet areas. TLOs were mainly associated with inflamed insulin-positive islets and their frequency was decreased in inflamed insulin-negative islets; they were not associated with insulin-negative, pseudo-atrophic islets lacking insulitis, and were not found in three donors with long disease duration (>30 years) who lacked insulitis and insulin-positive islets. All these data support a role for TLOs in the early stages of disease and in disease progression.

While most of the donors exhibited intermixed TLOs, four donors exhibited compartmentalised TLOs; two of the latter were at a particularly young age when diagnosed with diabetes (1.24–2.92 years). Disease duration was significantly shorter in these four donors compared with those with only intermixed T cells and B cells and those without TLOs, raising the possibility that compartmentalised TLOs are a feature of recent-onset type 1 diabetes and/or aggressive disease progression. Compartmentalised TLOs also exhibited structures closely resembling B cell follicles, as suggested by the presence of an FDC network surrounded by T cells and the presence of plasma cells and memory T cells. In addition, nuclear staining revealed areas of high and low cellular density within the B cell aggregates, consistent with the dark and light zones of germinal centres, respectively [37]. These data are consistent with published data showing a correlation between the presence of B cells in islet infiltrates in young donors with type 1 diabetes [38] and with the reported more aggressive disease progression in young individuals [39].

Tracer experiments performed in NOD mice confirmed that RFs in the TLOs were capable of transporting fluid, as described for LNs [8] and RIP-CXCL13 mice [17]. Autoantigen (insulin) and chemokines (CCL21) were detected within the RFs in NOD pancreases, supporting a conduit function for the RFs in the pancreatic TLOs. We were not able to detect chemokine or antigen in the RFs of human pancreatic TLOs. CCL21 was observed just on the surface of MEC79^+^ HEVs; this could be due to the differences between species, low expression level and/or quality of the samples.

TLOs were found in proximity to pancreatic ducts in samples from insulin-negative, pseudo-atrophic islets lacking insulitis and in the pancreas transplant biopsies, with an associated expression of insulin in ductal epithelial cells in the former case. We have previously observed insulin-positive ductal cells in pancreas transplant biopsies where there is recurrent type 1 diabetes [40]. It is plausible that insulin-positive cells in the ducts may represent regenerative or trans-differentiation events [40], which may attract an autoimmune infiltrate and recapitulate certain aspects of disease development, including TLO formation.

In conclusion, we demonstrate pancreatic TLOs at different stages of human type 1 diabetes and describe similarities and differences when compared with pancreatic TLOs in NOD mice. TLOs in the human pancreas with type 1 diabetes appear at sites of active autoimmunity but are not detected once the destructive process has run its course. These data are consistent with studies in NOD mice showing that TLOs disappear once beta cells, the antigen source, perish [15]. Importantly, the presence of TLOs in preclinical organ donors exhibiting insulitis suggests that they form before development of clinical symptoms and supports their role in disease progression. Further studies should refine the characterisation of immune subtypes within the TLOs and investigate the presence of autoantigen-specific plasma cells and T cells, as these may vary by stage and severity.

## Supplementary information

ESM (PDF 31.2 mb)

## Data Availability

Further information about the data are available from the corresponding author upon request.

## Abbreviations

aAb: Autoantibody
BM: Basement membrane
EADB: Exeter Archival Diabetes Biobank
ECM: Extracellular matrix
FDC: Follicular dendritic cell
FRC: Fibroblastic reticular cell
FOXP3: Forkhead box P3
GADA: GAD aAb
HEV: High endothelial venule
IAA: Insulin aAb
IA-2A: Tyrosine phosphatase-related islet antigen 2 aAb
LN: Lymph node
nPOD: Network for Pancreatic Organ Donors with Diabetes
PDGFrβ: Platelet-derived growth factor receptor β
PLM: Pan-laminin
RF: Reticular fibre
SPK: Simultaneous pancreas and kidney
TLO: Tertiary lymphoid organ
ZnT8A: Zinc transporter 8 aAb
