# Supplementary material for: Identification and characterisation of tertiary lymphoid organs in human type 1 diabetes
Source: Diabetologia. 2021 Apr 29;64(7):1626–41. doi: 10.1007/s00125-021-05453-z (PMC8187221; doi:10.1007/s00125-021-05453-z)
Supplement: Supplementary file 1 — (PDF 31.2 mb) [file 125_2021_5453_MOESM1_ESM.pdf]

ELECTRONIC SUPPLEMENTARY MATERIAL

ESM Results

Characterization of RFs in TLOS of NOD mice.

We conducted an in-depth characterization of the RFs in TLOs of NOD mice using a broad repertoire of antibodies directed against specific BM and interstitial matrix (IM) components of RFs [1, 2] (ESM Table 1). These novel analyses show that RFs of NOD pancreatic TLOs stain positively for fibrillar collagen types I and III (ESM Fig. 8) and microfibrillar molecules. The latter include: collagen type VI, ERTR7, fibrillin-2, matrilin-2, fibronectin and tenascin-C. BM proteins identified were collagen type IV, perlecan, agrin, nidogen-2, as well as several laminin chains - laminin-α2, -α3, -α4, -α5, -β1, -β2, -γ1, -γ2 (ESM Fig. 8), which can form different functional heterotrimers (laminins 211, 322, 411, 421, 511 and 521).

Conduit function of RFs

Since RFs of LNs can act as conduits for the rapid transport of soluble, low molecular weight molecules such as chemokines and antigens [1, 3], we investigated whether the RFs of pancreatic TLO´s have a similar function. Low molecular weight FITC-Dextran was injected i.v. into the tail vein of 12 week-old NOD mice and the pancreases were excised and analysed microscopically. Strong tracer accumulation was detected in blood vessels throughout the pancreas and in RFs of TLOs (ESM Fig. 9a), suggesting that RFs can transport molecules from the circulation into the pancreas.

Direct staining of NOD mouse pancreatic sections also revealed the CCL21 chemokine, important for the recruitment of T cells to LNs [4], in the core of RFs, on the surface of HEVs, in blood vessels at the inflammatory front, and on lymphatic endothelium (ESM Fig. 9b). Similarly, inflamed islets of donors with type 1 diabetes and insulitis showed CCL21 staining

in the blood vessels of the islets and on HEVs (ESM Fig. 9c), but not in the RFs (ESM Fig. 9c). Staining for the insulin autoantigen revealed a gradient of decreasing staining intensity in the core of RFs from the front of the inflammation towards the periphery of the insulitis region in NOD samples only (ESM Fig. 9d). Taken together these data suggest that RF structure is similar in mouse and human TLOs and, albeit limited to chemokine staining in human samples, that they may have a conduit function as in LNs.

Tracer accumulation, while occurring in the pancreas, was also observed in both the reticular fibres and blood vessel lumens of the spleen and LNs, but was restricted to blood vessel lumens in the brain and skeletal muscle, which is consistent with other similar studies [1, 5, 6]. Likewise, a CCL21 immunofluorescence staining was observed in the RF conduits of LNs, as also reported previously by others [6]

ESM Table 1 List of antibodies employed

Molecule	Antibody name/clone	Dilution	Reactivity with mouse/human	Reference/source
**BM molecules**				
PLM	455	1/500	Mouse/human	[7]
Laminin α2	401	1/200	Mouse/human	[7]
Laminin α3		1/400	Mouse/human	Gift from T. Sasaki, Oita University, Japan
Laminin α4	377	1/200	Mouse/human	[7]
Laminin α5	405	1/200	Mouse	[7]
	4C7	Supernatant fraction	Human	[7]
Laminin β1	3A4	Supernatant fraction	Mouse	[7]
Laminin β2	489	1/500	Mouse/human	[7]
Laminin γ1	3E10	Supernatant fraction	Mouse/human	[7]
Laminin γ2		1/400	Mouse	Gift from T. Sasaki, Oita University, Japan
Laminin γ3		1/200	Mouse	Gift from T. Sasaki, Oita University, Japan
Collagen IV		1/200	Mouse/human	Millipore/Chemicon (cat. no. AB756P)/Germany
Perlecan	A716	1/200	Mouse/human	[7]
Agrin		1/200	Mouse	[7]
Nidogen-1		1/200	Mouse/human	[7]
Nidogen-2		1/200	Mouse/human	[7]
**Interstitial matrix molecules**				
Collagen I		1/200	Mouse	Chemicon (cat. no. AB765P)/Germany
Collagen III		1/200	Mouse/human	Southern Biotech (cat. no. 1330-01)/Biozol/Germany
Collagen VI		1/1000	Mouse/human	Fitzgerald (cat. no. 70R-CR009X)/Biozol/Germany
Fibronectin		1/200	Mouse/human	[7]
Tenascin-C	MT4	Supernatant fraction	Mouse	[7]
Matrillin-2		1/200	Mouse	R&D Systems (cat. no. AF3234)/Germany
Fibrillin-1		1/200	Mouse/human	[7]
Fibrillin-2		1/200	Mouse/human	[7]
ERTR7		1/200	Mouse/human	Dianova/BMA (cat. no. T-2109)/Germany
**ECM receptors and cell markers**				
Podoplanin	8.1.1	Supernatant fraction	Mouse	[7]
		1/50	Human	Abcam (cat. no. ab10274)/Germany
PDGFrβ	APB5	1/200	Mouse	Thermo Fisher Scientific (cat. no. 14-1402-82)/Germany
		1/50	Human	Cell Signaling (cat. no. 3169S)/Germany
CD45	30G12	Supernatant fraction	Mouse	[7]

	L3B12	Supernatant fraction	Human	[7]
CD3		1/50	Mouse/human	Abcam (cat. no. ab16669)/Germany
B220	RA3-6B2	1/100	Mouse	BD Pharmingen (cat. no. 553084)/Germany
CD20		1/100	Human	Abcam (cat. no. ab 9475)/Germany
CD21		1/100	Mouse	BD Pharmingen (cat. no. 558768)/Germany
			Human	Abcam (cat. no. 75985)/Germany
MECA32 antigen	MECA32	1/1000	Mouse	[8]
MECA79		1/50	Mouse/human	BD Pharmingen (cat. no. 553863)/Germany
CD31	Rb10	Supernatant fraction	Human	http://www.hcdm.org/MoleculeInformation/tabid/54/Default.aspx
		1/200	Mouse	BD Pharmingen (cat. no. 550274)/Germany
Insulin		1/400	Mouse/human	DAKO (cat. no. A0564)/Germany
**Chemokine**				
CCL21		1/200	Mouse	R&D Systems (cat. no. AF457)/Germany

ESM Fig. 1 An example for primary antibody validation on a human LN section. Immunofluorescence staining for MECA79 marks only HEVs (arrows) but CD31 visualizes all blood vessels. Note the thick endothelial wall of HEV´s (MECA79^{+}/CD31^{+}) compared to the wall of capillaries (only CD31^{+}). Negative control shows the specificity of secondary antibodies. Scale bars, 100 µm.

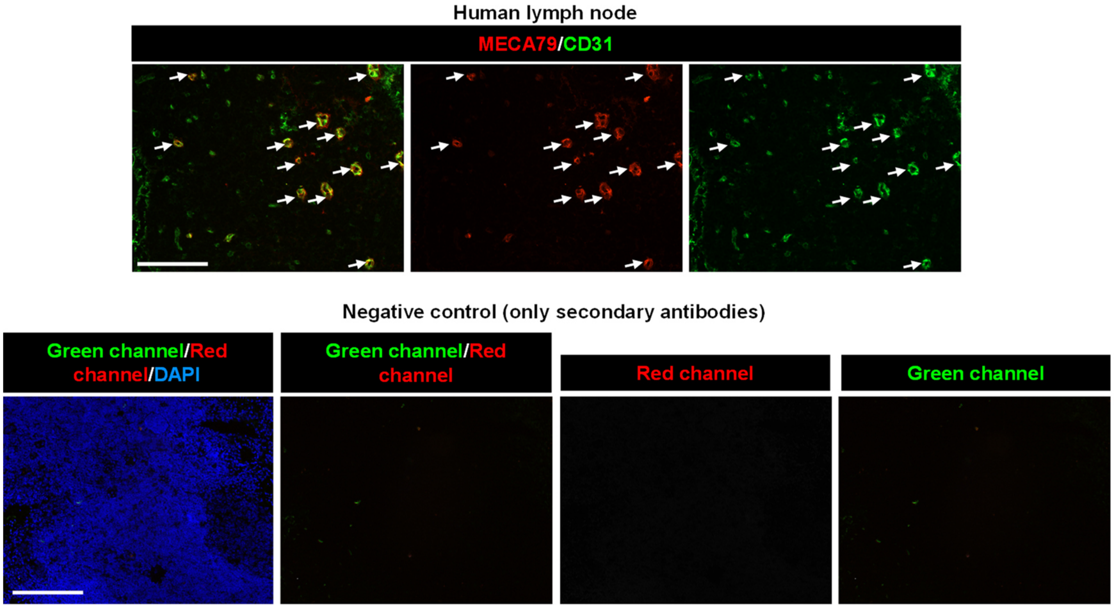

ESM Fig. 2 Immunofluorescence staining for PLM allows identification of pancreatic islets. (a) Insulin-positive islets co-stained with PLM and (b) insulin-negative/glucagon-positive islets are shown. PLM marks the peri-islet BM; endothelial BMs of blood vessels and the acinar BMs. Scale bars, 100 µm.

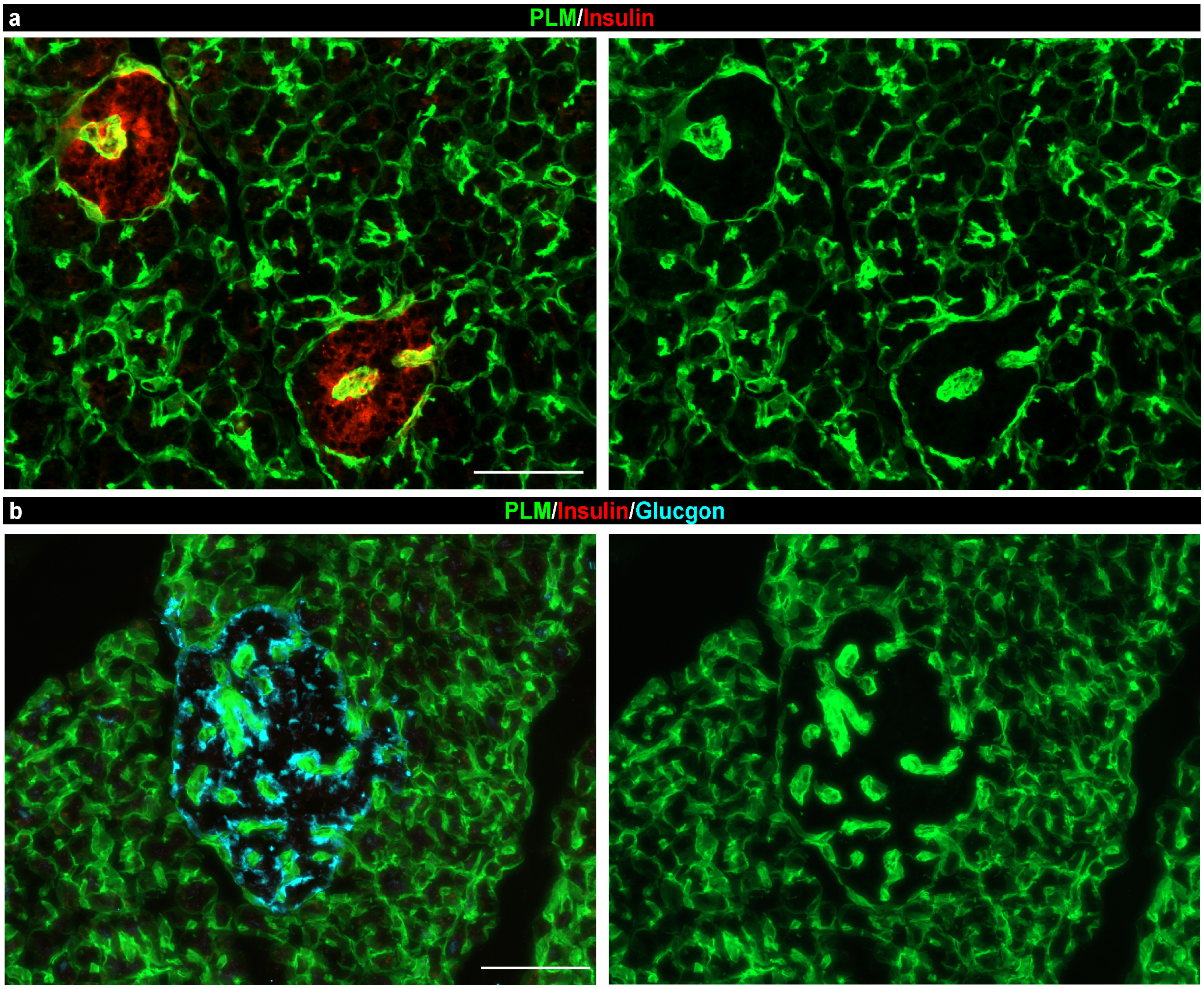

ESM Fig. 3 Correlation between the frequency of TLOs with age of type 1 diabetes onset (top panel) and with disease duration (bottom panel) in donors with TLOs. Frequency data were those for islets in phase 1 (insulin^{+} islets with inulitis) and 2 (insulin^{-} islets with insulitis). Each circle denotes an individual donor.

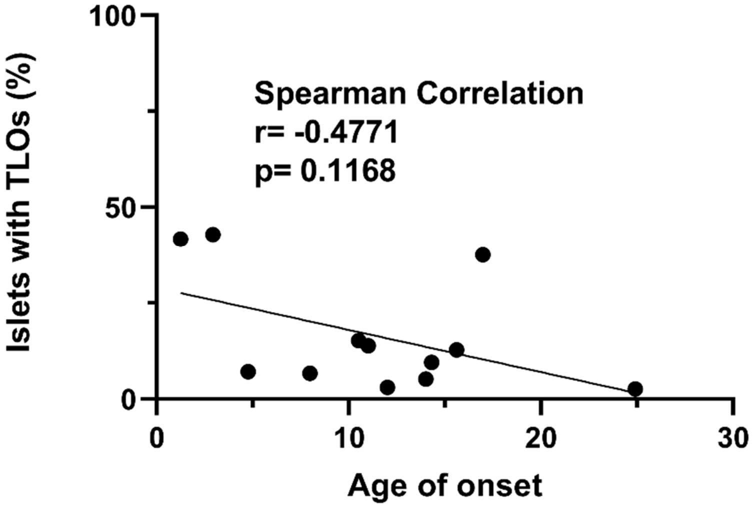

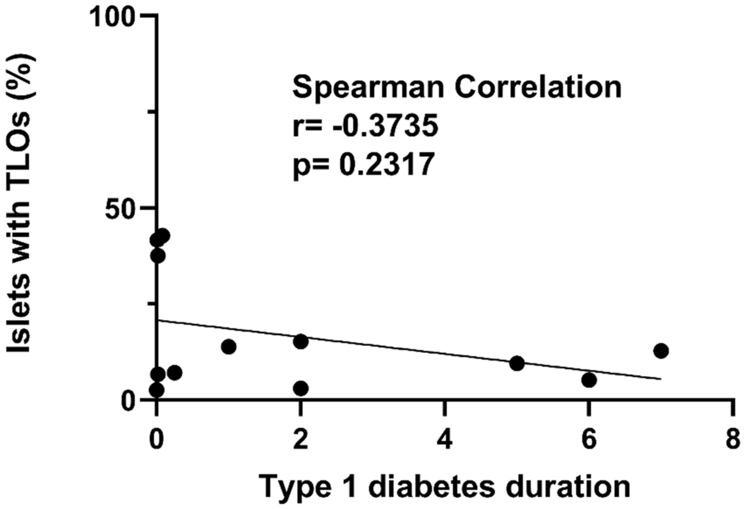

ESM Fig. 4 (A-D) T and B cell compartments of TLOs in pancreas sections of type 1 diabetes donors: no. E124 (A), no. E308 (B), no. 6362 (C) and no. SC115 (D) shown at low and high magnifications. The compartmentalized TLOs were mostly associated with insulin^{+} islets (A-C), and rarely with insulin^{-} negative islets (D).

(A) Organ donor no. E124B (age at onset: 16.98 years; disease duration: 0.02 years). Immunofluorescence staining reveals T (CD3$^+$) and B (CD20$^+$) cell compartments and HEVs (MECA79$^+$, arrow) (i). FDCs of the B cell compartment (CD20$^+$) are visualized by CD21$^+$ staining (ii) . Anti-CD45 marks all leucocytes; anti-collagen VI (Coll VI) marks RFs of the T cell (arrowhead) and B cell (star) compartment (iii). DAPI marks all nuclei. Scale bars 100 µm (i) or 50 µm (ii-iii); is, islet.

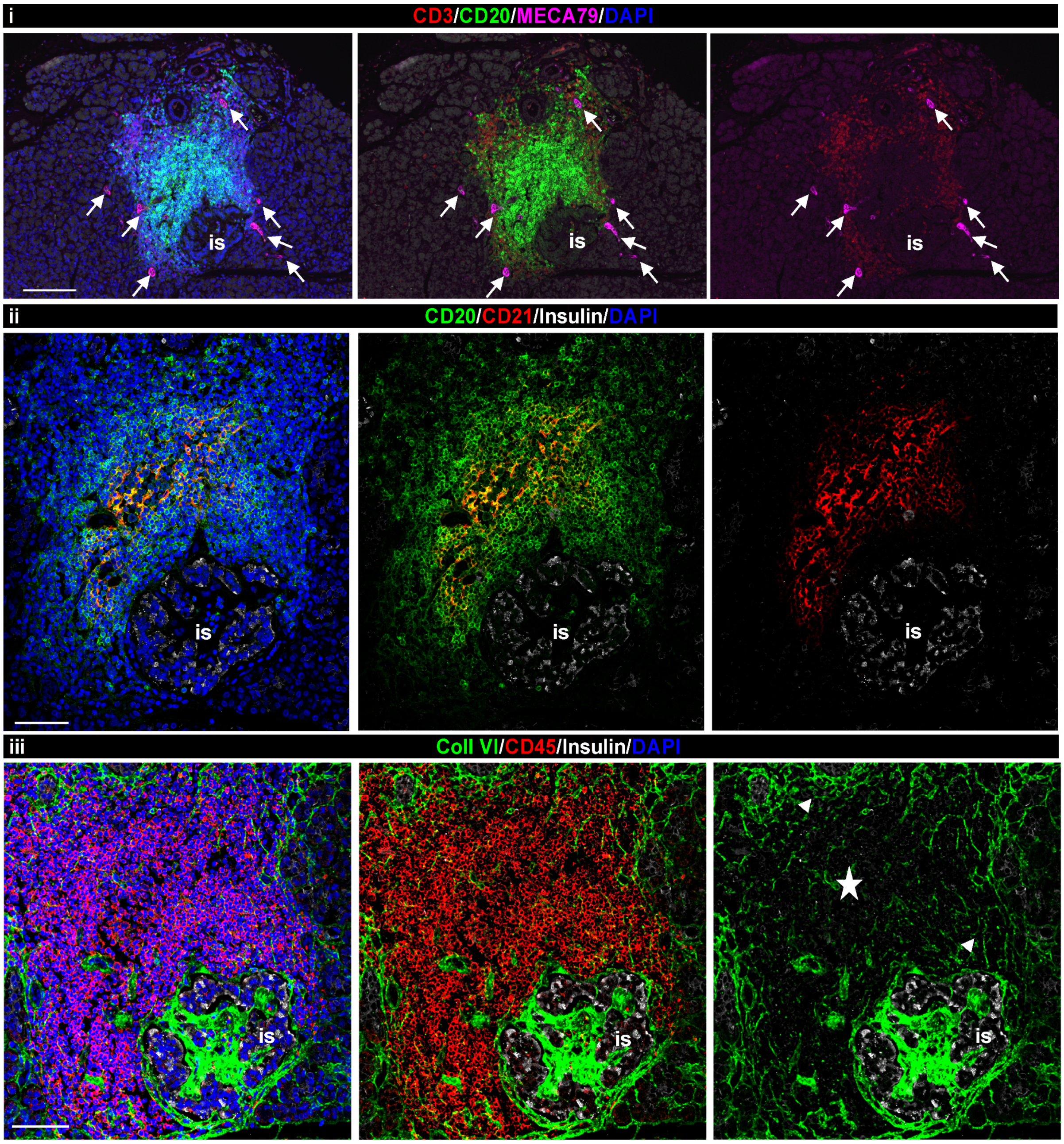

(B) Organ donor no. E308 (age at onset: 2.92 years; disease duration: 0.08 year). Immunofluorescence staining to show T ($CD3^+$) and B ($CD20^+$) cell compartments and FDCs ($CD21^+$) (i-ii). Arrowheads mark the collagen IV^+ RFs in the $CD3^+$ T cell zone, while star labels the $CD20^+$ B cell zone with sparse RFs (iii). Boxed area is shown at higher magnification. DAPI marks nuclei. Scale bars 100 µm.

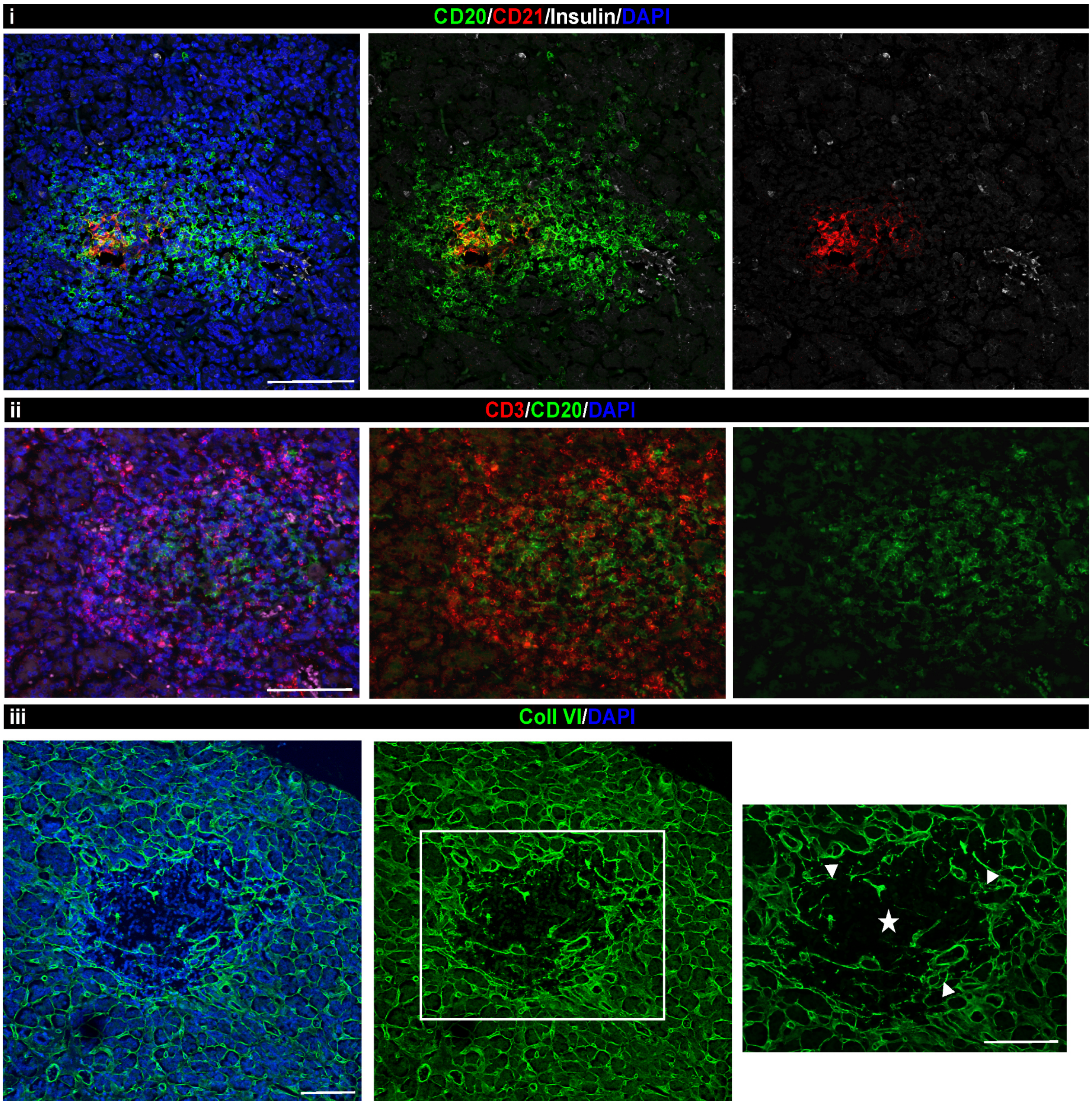

(C) Organ donor no. 6362 (age at onset: 24.9 years; disease duration: 0 year). Immunofluorescence staining for PLM to mark the BMs of the exocrine and endocrine regions, CD45 to mark leucocytes and insulin to label beta cells. Boxed areas (1, 2) are shown at higher magnifications. Scale bars, 100 µm.

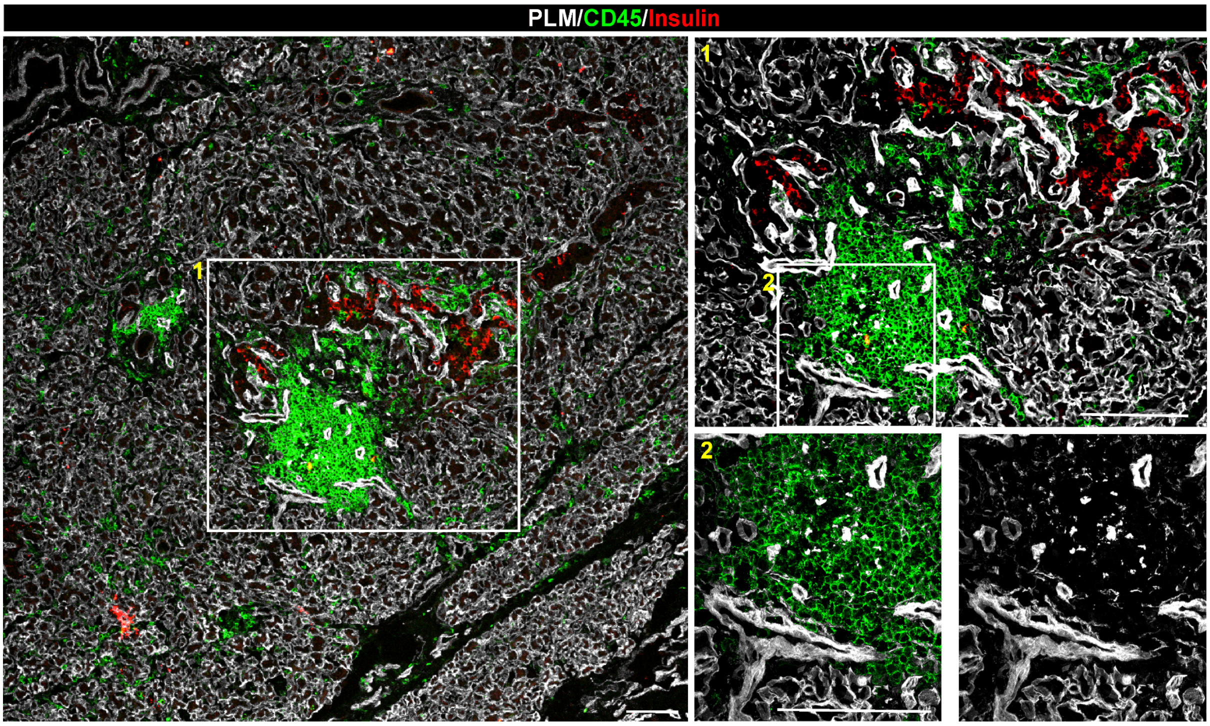

(D) Organ donor no. SC 115 (age at onset: 1.24 year; disease duration: 0.01 year). Whole image scan of a pancreatic section stained for insulin to mark beta cells, for glucagon to mark alpha cells and haematoxyline to mark nuclei. Boxed area is shown at higher magnifictaion (i). Parallel sections were stained for CD20 to mark B cell zone and for CD21 to mark FDCs (ii) Immunofluorescence staining for $CD3^+$ T cells and $MECA79^+$ HEVs (ii, arrows), or (d) collagen type VI and III (iv); DAPI mark all nuclei. Arrowheads in (iv) mark RFs in the T cell zone. Scale bars, 1 mm (i); 100 µm (inset in i, ii-iv).

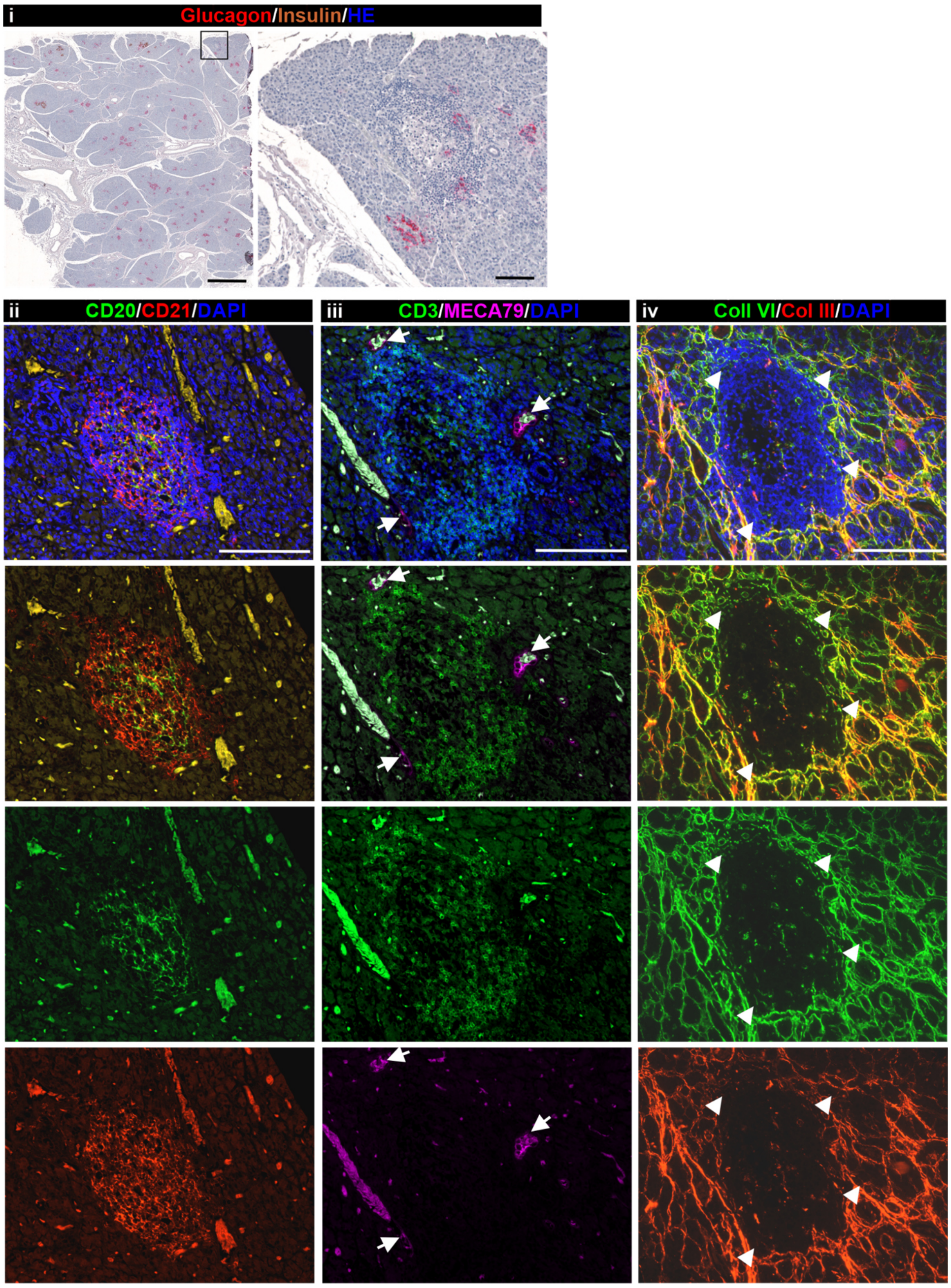

ESM Fig. 5 Association of TLOs with the presence of autoantibodies. Comparing donors with TLOs with donors without TLOs we found no significant differences in the positivity rates for each aAb (excluding IAA, not tested) and for multiple aAbs by Fisher´s exact test.

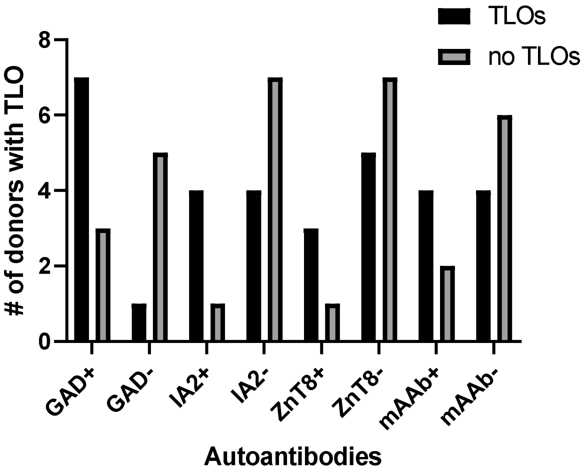

ESM Fig. 6 RF network associated with T and B cell compartments in mouse LNs. (a) Triple immunofluorescence staining reveals the CD3$^+$ T cell zone and CD20$^+$ B cell zone (star) and laminin α4$^+$ RFs. Note the thick BM of HEVs (arrowhead). (b) PLM staining shows the RFs in T cell (arrowheads) and B cell zone (star) and PDGFrβ staining labels the FRCs associated with RFs. Note the denser network of RFs in the T cell zone compared to the B cell zone. Scale bars, 100 µm. c, capsule; rf, reticular fibres.

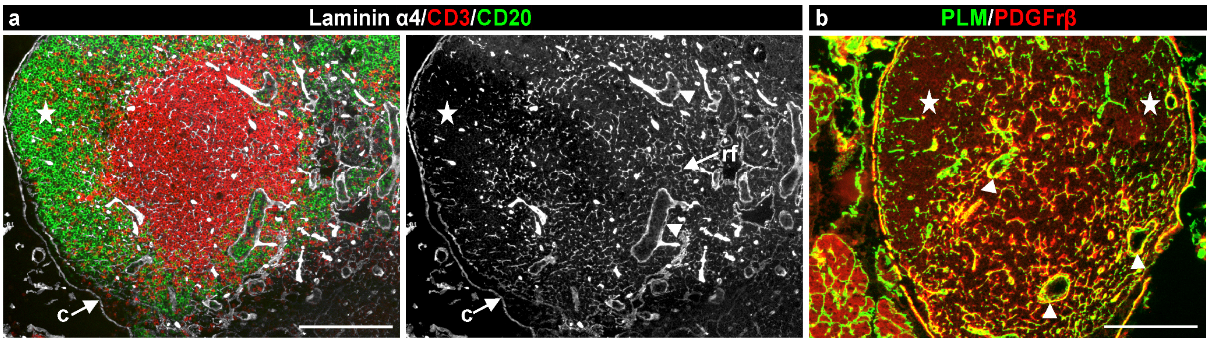

ESM Fig. 7 Electron micrograph of the main Figure 5f with grid. Scale bar 5 μm.

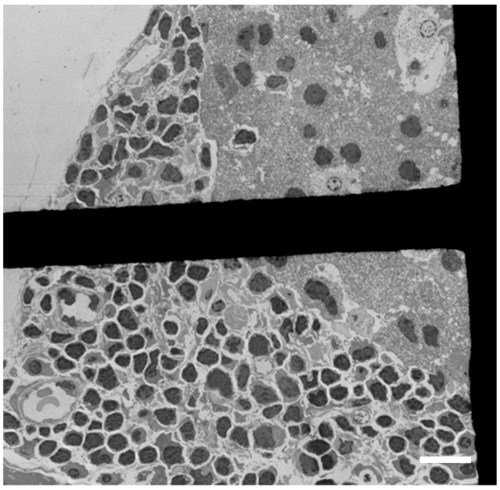

ESM Fig. 8 Extracellular matrix composition of reticular fibres in the pancreatic TLOs of NOD mice. Representative images of inflamed islets stained for basement membrane and interstitial matrix proteins and CD45, a general leucocyte marker. Scale bars, 100 µm; is, islet; du, duct; ac, acini.

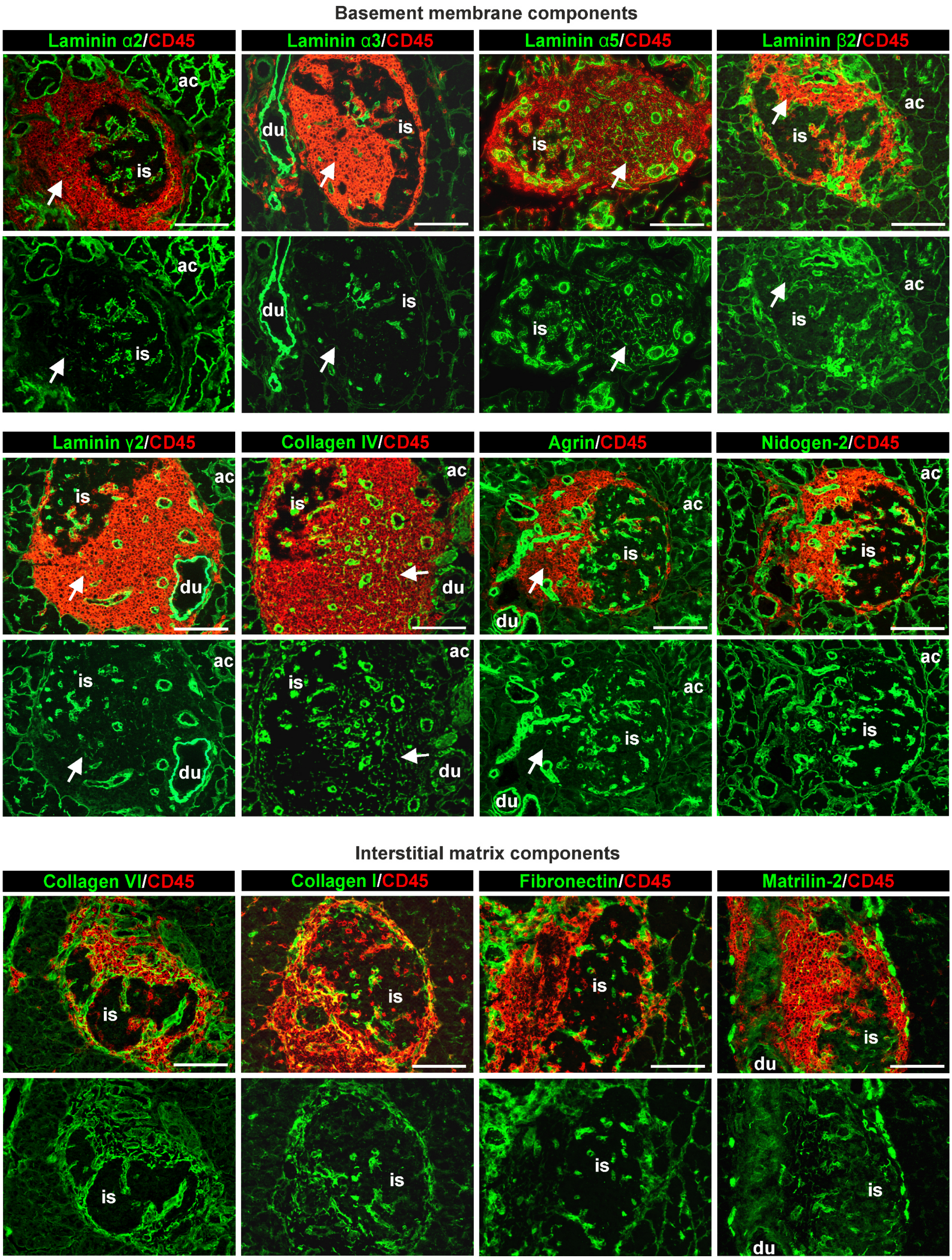

ESM Fig. 9 Conduit function of RFs. (a) I.v. injected 40 kDa FITC-dextran (FITC-DEXT) is detected in NOD pancreas TLOs in the inner core of RFs; PLM staining marks the outer layer of RFs. Immunofluorescence staining for CCL21 shows the presence of chemokines in the blood vessels and HEVs of inflamed (b) mouse and (c) human pancreatic islets; CCL21 was also detect in the RFs and lymphatic vessels of NOD islets (b). Arrow indicates RFs and arrowhead marks HEVs. (d) PLM, insulin and CD45 triple immunofluorescence staining showing insulin within the RFs (arrows in inserts); boxed area is shown at higher magnification. Scale bars, 50 µm; 12.5 µm (d; area shown at higher magnification); lv, lymphatic vessels; is, islets.

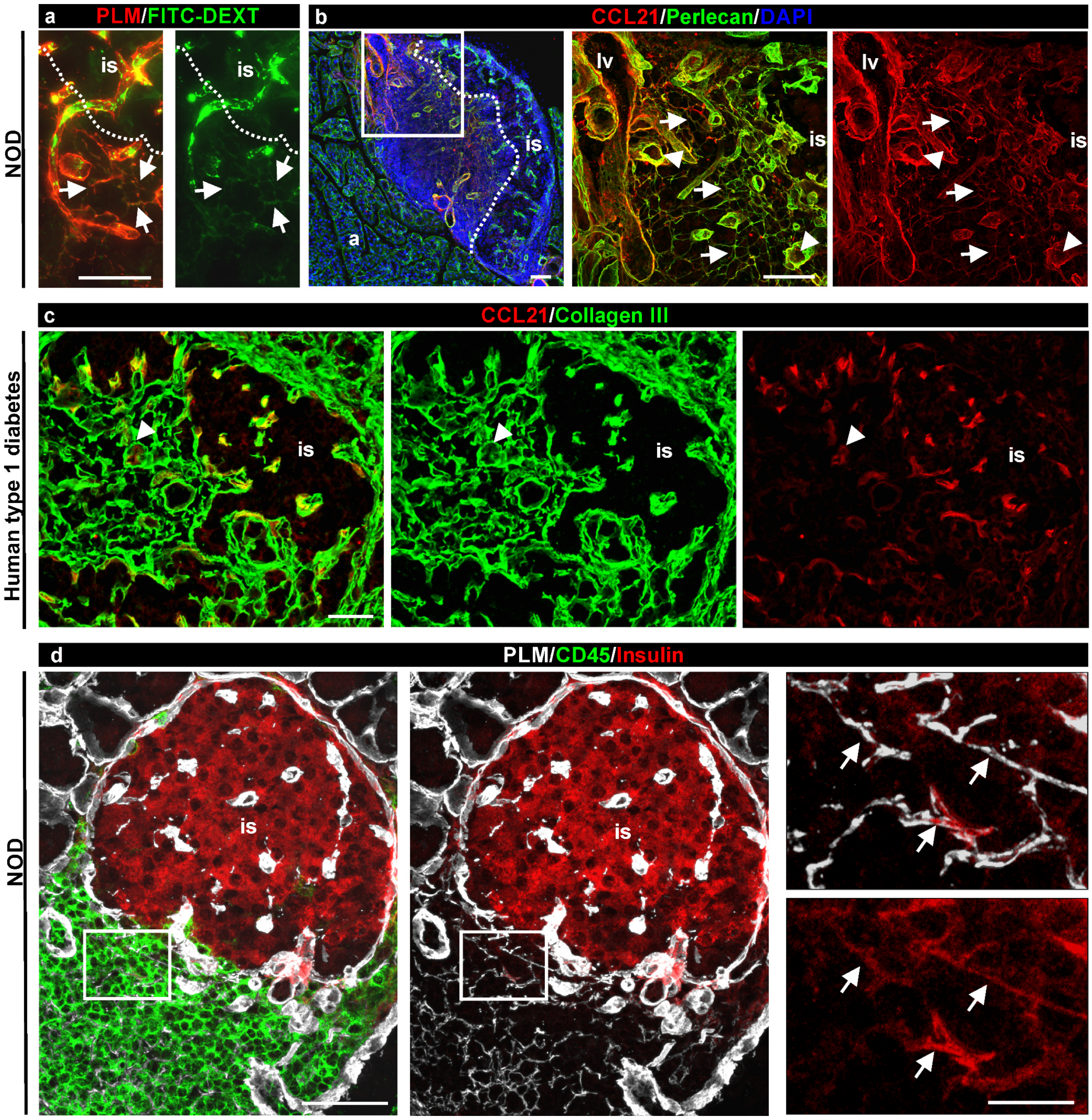

REFERENCES

[1] Sixt M, Kanazawa N, Selg M, et al. (2005) The conduit system transports soluble antigens from the afferent lymph to resident dendritic cells in the T cell area of the lymph node. Immunity 22(1): 19-29. 10.1016/j.immuni.2004.11.013

[2] Lokmic Z, Lammermann T, Sixt M, Cardell S, Hallmann R, Sorokin L (2008) The extracellular matrix of the spleen as a potential organizer of immune cell compartments. Semin Immunol 20(1): 4-13. 10.1016/j.smim.2007.12.009

[3] Gretz JE, Norbury CC, Anderson AO, Proudfoot AE, Shaw S (2000) Lymph-borne chemokines and other low molecular weight molecules reach high endothelial venules via specialized conduits while a functional barrier limits access to the lymphocyte microenvironments in lymph node cortex. J Exp Med 192(10): 1425-1440. 10.1084/jem.192.10.1425

[4] Schulz O, Hammerschmidt SI, Moschovakis GL, Forster R (2016) Chemokines and Chemokine Receptors in Lymphoid Tissue Dynamics. Annu Rev Immunol 34: 203-242. 10.1146/annurev-immunol-041015-055649

[5] Nolte MA, Belien JA, Schadee-Eestermans I, et al. (2003) A conduit system distributes chemokines and small blood-borne molecules through the splenic white pulp. J Exp Med 198(3): 505-512. 10.1084/jem.20021801

[6] Link A, Hardie DL, Favre S, et al. (2011) Association of T-zone reticular networks and conduits with ectopic lymphoid tissues in mice and humans. Am J Pathol 178(4): 1662-1675. 10.1016/j.ajpath.2010.12.039

[7] Korpos E, Kadri N, Kappelhoff R, et al. (2013) The peri-islet basement membrane, a barrier to infiltrating leukocytes in type 1 diabetes in mouse and human. Diabetes 62(2): 531-542. 10.2337/db12-0432

[8] Hallmann R, Mayer DN, Berg EL, Broermann R, Butcher EC (1995) Novel mouse endothelial cell surface marker is suppressed during differentiation of the blood brain barrier. Dev Dyn 202(4): 325-332. 10.1002/aja.1002020402
